# Coating and Corruption of Human Neutrophils by Bacterial Outer Membrane Vesicles

**DOI:** 10.1128/spectrum.00753-22

**Published:** 2022-08-24

**Authors:** Marines du Teil Espina, Yanyan Fu, Demi van der Horst, Claudia Hirschfeld, Marina López-Álvarez, Lianne M. Mulder, Costanza Gscheider, Anna Haider Rubio, Minke Huitema, Dörte Becher, Peter Heeringa, Jan Maarten van Dijl

**Affiliations:** a Department of Medical Microbiology, University of Groningen, University Medical Center Groningen, Groningen, The Netherlands; b Institute for Microbiology, Ernst-Moritz-Arndt-University Greifswald, Greifswald, Germany; c Department of Pathology and Medical Biology, University Medical Center Groningen, University of Groningen, Groningen, The Netherlands; Griffith University

**Keywords:** OMVs, *Porphyromonas gingivalis*, neutrophils, myeloperoxidase, citrullination, gingipains, LL-37, outer membrane vesicles

## Abstract

Porphyromonas gingivalis is a keystone oral pathogen that successfully manipulates the human innate immune defenses, resulting in a chronic proinflammatory state of periodontal tissues and beyond. Here, we demonstrate that secreted outer membrane vesicles (OMVs) are deployed by P. gingivalis to selectively coat and activate human neutrophils, thereby provoking degranulation without neutrophil killing. Secreted granule components with antibacterial activity, especially LL-37 and myeloperoxidase (MPO), are subsequently degraded by potent OMV-bound proteases known as gingipains, thereby ensuring bacterial survival. In contrast to neutrophils, the P. gingivalis OMVs are efficiently internalized by macrophages and epithelial cells. Importantly, we show that neutrophil coating is a conserved feature displayed by OMVs of at least one other oral pathogen, namely, Aggregatibacter actinomycetemcomitans. We conclude that P. gingivalis deploys its OMVs for a neutrophil-deceptive strategy to create a favorable inflammatory niche and escape killing.

**IMPORTANCE** Severe periodontitis is a dysbiotic inflammatory disease that affects about 15% of the adult population, making it one of the most prevalent diseases worldwide. Importantly, periodontitis has been associated with the development of nonoral diseases, such as rheumatoid arthritis, pancreatic cancer, and Alzheimer’s disease. Periodontal pathogens implicated in periodontitis can survive in the oral cavity only by avoiding the insults of neutrophils while at the same time promoting an inflamed environment where they successfully thrive. Our present findings show that outer membrane vesicles secreted by the keystone pathogen Porphyromonas gingivalis provide an effective delivery tool of virulence factors that protect the bacterium from being killed while simultaneously activating human neutrophils.

## INTRODUCTION

Bacterial pathogens play a hide-and-seek game with the human immune defenses, employing a plethora of secreted virulence factors. A very effective mechanism of virulence factor delivery has evolved in Gram-negative bacteria, in which membrane vesicles loaded with a cargo of proteins and lipopolysaccharides are pinched off from the outer membrane. These so-called outer membrane vesicles (OMVs) subsequently interact with cells and tissues of the human host, ultimately allowing the bacteria to colonize particular niches (1–3). OMVs even serve as long-range missiles that break barriers to acquire resources from the host, while the bacteria stay at a safe distance from potentially lethal immune responses (3). In a recent study, we have shown that the Gram-negative bacterial pathogen Porphyromonas gingivalis effectively neutralizes human innate immune defenses by employing an OMV-associated virulence factor, the Porphyromonas peptidyl-arginine deiminase (PPAD) that citrullinates both bacterial and human host proteins through the deamination of arginine residues (4–7). These observations are important, because P. gingivalis is a primary causative agent of periodontitis (8), a chronic inflammatory condition severely affecting 10 to 15% of the adult population worldwide, eventually leading to alveolar bone resorption and tooth loss (9–11). Moreover, periodontitis is implicated in many chronic and highly debilitating diseases ranging from rheumatoid arthritis to pancreatic and orodigestive cancers, as well as Alzheimer’s disease (12–15).

In addition to PPAD, P. gingivalis OMVs carry potent cysteine proteases known as the gingipains RgpA, RgpB, and Kgp, which are generally regarded as the major virulence factors of P. gingivalis (8, 16). These gingipains effectively cleave bacterial and human proteins at arginine (RgpA and RgpB) or lysine (Kgp) residues (8). Consequently, the gingipains are fundamental to many pathogenic activities, in particular the interference with host immune responses (16–19). Interestingly, since PPAD preferentially citrullinates terminal arginine residues, as generated by the action of RgpA and RgpB, terminally citrullinated peptides will be presented to the host (7). This is especially relevant in view of the previously documented association of periodontitis with the common autoimmune disorder rheumatoid arthritis, which is characterized by autoantibodies against citrullinated proteins (14, 20).

Neutrophils represent the most abundant leukocytes (>95%) recruited to the gingival crevice in response to the action of periodontal pathogens, such as P. gingivalis (18, 21, 22). These professional phagocytes form a formidable defense line that prevents bacterial access to deeper-seated tissues (22). In particular, the neutrophils eliminate invading pathogens through phagocytosis and intracellular killing, the formation of neutrophil extracellular traps (NETs) that capture pathogens extracellularly, and the release of granule-derived proteins and peptides with antimicrobial activities (21, 23–25). The most abundant granule enzyme is the myeloperoxidase (MPO), which generates potent antimicrobial oxidants (23, 26). A highly effective granule-derived cationic antimicrobial peptide (CAMP) that is important for periodontal health is LL-37, which is in fact the only cathelicidin-derived peptide known in humans (27). Despite these generally effective defense mechanisms, P. gingivalis is able to corrupt the neutrophil responses, thereby avoiding its elimination and enhancing inflammatory responses that ultimately contribute to the destruction of periodontal tissues. Importantly, both PPAD and the gingipains are key elements in the P. gingivalis strategy to bypass the neutrophil defense line (4, 18, 21, 28).

**FIG 1:**
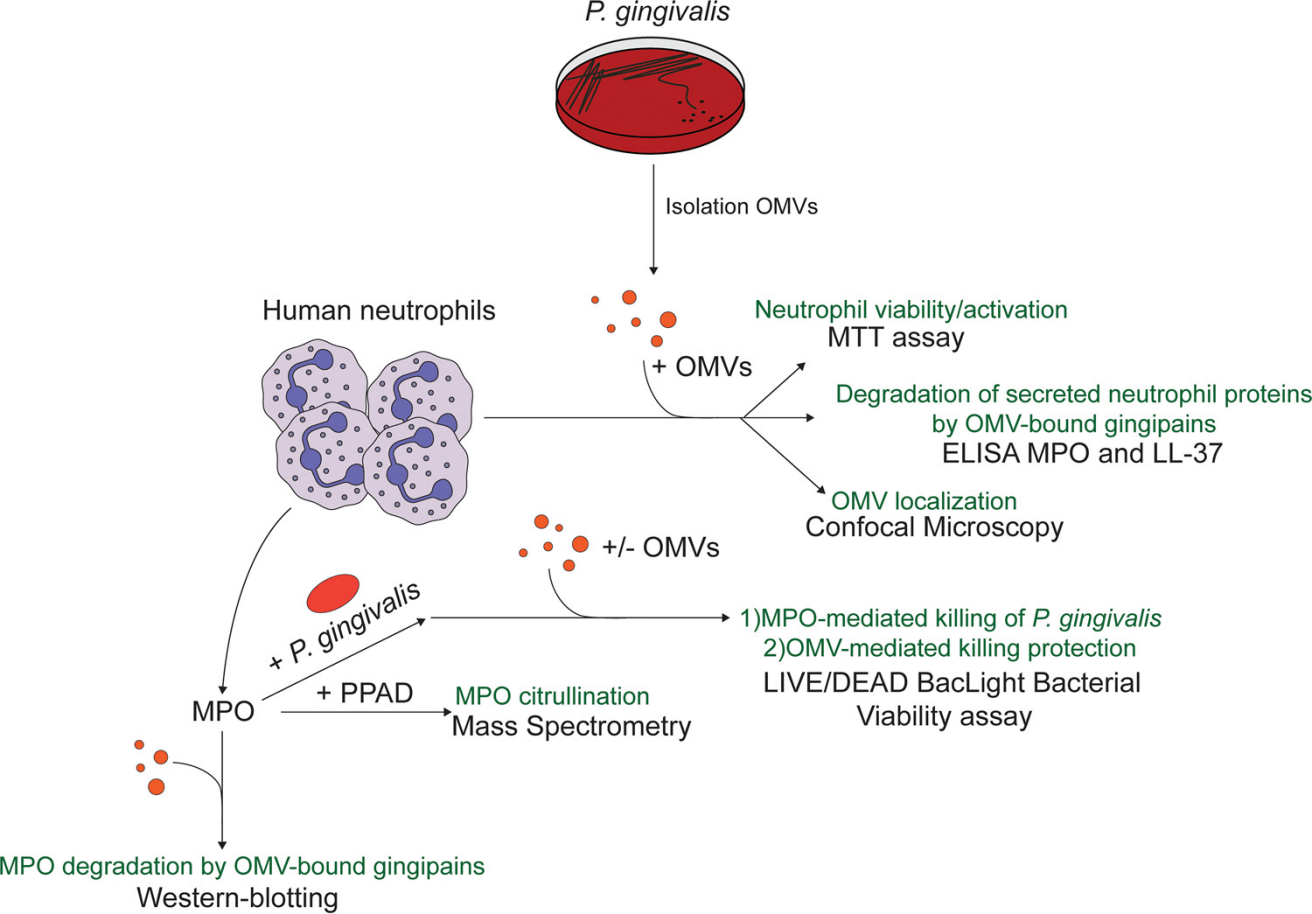
Schematic representation of the design of studies on the interactions between P. gingivalis outer membrane vesicles (OMVs) and human neutrophils. Human neutrophils were challenged with OMVs of P. gingivalis. Subsequently, the viability of the neutrophils was assessed by measuring reduction of 3-[4,5-dimethylthiazol-2-yl]-2,5-diphenyl tetrazolium bromide (MTT), the interaction of neutrophils and OMVs was assessed by microscopy, and the turnover of myeloperoxidase (MPO) and the antimicrobial peptide LL-37 in neutrophil supernatants was measured by enzyme-linked immunosorbent assays (ELISAs). In addition, the bactericidal activity of purified MPO was assayed using LIVE/DEAD staining, possibly citrullination by mass spectrometry (MS), and turnover by Western blotting. PPAD, Porphyromonas peptidyl-arginine deiminase.

Notably, while OMVs of P. gingivalis are known to carry the major virulence factors of this pathogen, it was thus far poorly understood how these delivery vehicles are deployed in the conflict between the pathogen and its prime opponent, the neutrophil. Therefore, the aim of this study was to determine how the OMVs of P. gingivalis interact with neutrophils with particular focus on the roles of OMV-borne PPAD and gingipains on the activity of the granule-derived MPO and LL-37. By studying the interactions between P. gingivalis OMVs and human neutrophils as outlined in Fig. 1, we show that OMVs specifically coat the neutrophil, bind to NETs, inactivate MPO, and degrade LL-37. To verify whether neutrophil coating by OMVs is unique to P. gingivalis, we employed another major oral pathogen associated with periodontitis and systemic diseases, namely Aggregatibacter actinomycetemcomitans (29). In previous studies, it was shown that OMVs of A. actinomycetemcomitans were internalized into HeLa cells and human gingival fibroblasts (30). Here, we show that OMVs of A. actinomycetemcomitans also have the ability to coat human neutrophils. We therefore propose that specific neutrophil coating by OMVs may be a conserved feature among periodontal pathogens.

## RESULTS

### OMVs of P. gingivalis coat the membrane of neutrophils without affecting their viability.

**FIG 2:**
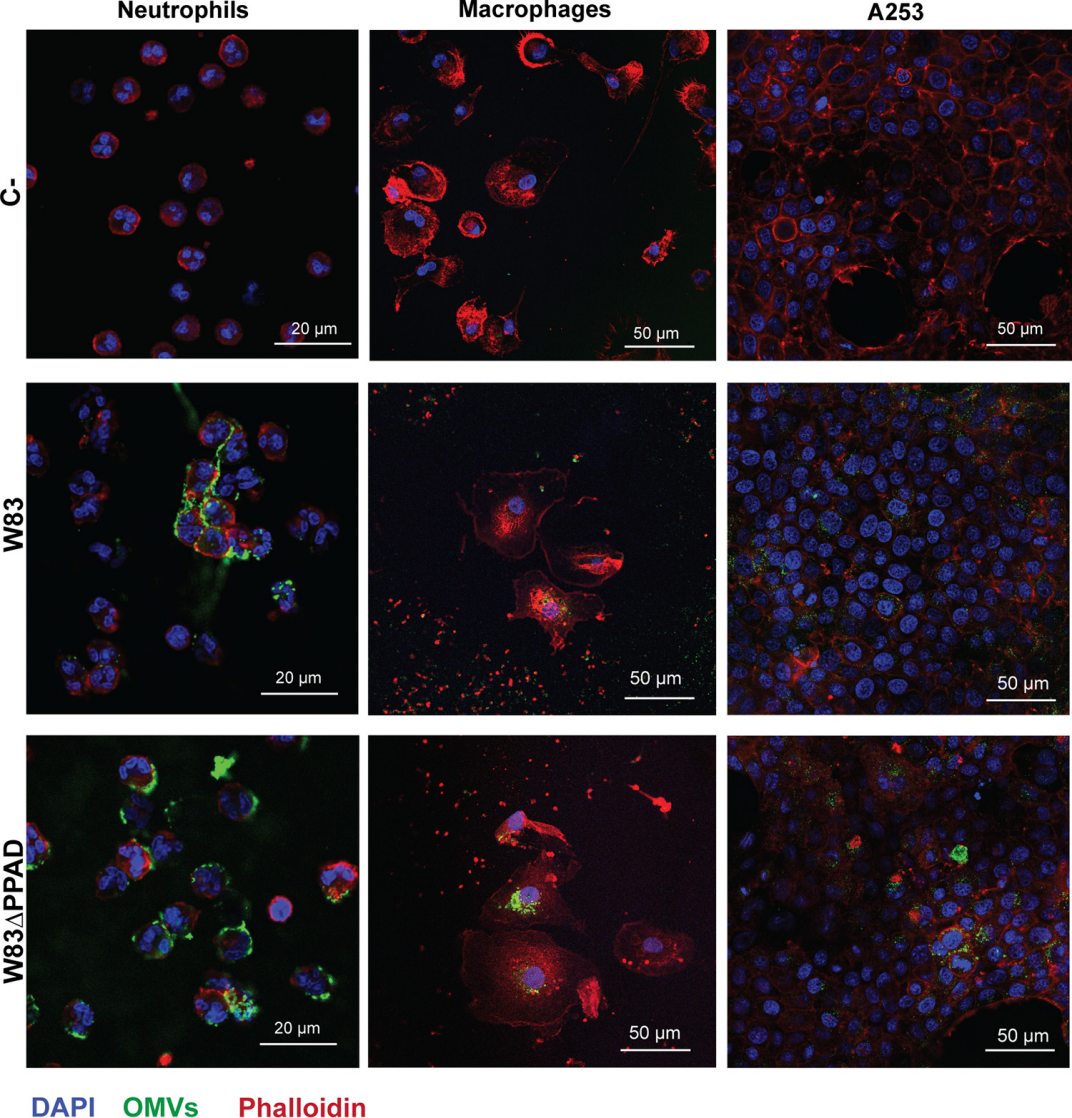
OMVs of P. gingivalis form a coat on the neutrophil surface while they are internalized by other cell types. Shown are representative confocal microscopy overlay images of neutrophils, macrophages, and A253 cells challenged with 5 μg of OMVs from P. gingivalis W83 or W83ΔPPAD. Unchallenged neutrophils, macrophages, and A253 cells were used as a control (C-). The images show OMV binding to the neutrophil’s surface and internalization by macrophages and A253 cells. 4′,6-Diamidino-2-phenylindole (DAPI) was used to stain the neutrophils’ nuclei (blue) and phalloidin-tetramethyl-rhodamine B isothiocyanate (TRITC) was used to stain actin (red). OMVs were labeled with P. gingivalis-specific polyclonal rabbit antibodies and secondary goat anti-rabbit antibodies labeled with Alexa Fluor 488 (green). Bars mark 20 or 50 μm as indicated in each panel. The individual images used to create the overlays are presented in Fig. S3.

To visualize the interactions of P. gingivalis OMVs with human neutrophils, we challenged the neutrophils for 90 min with different amounts of OMVs (1 and 5 μg) purified from the growth medium of P. gingivalis W83 or the PPAD-deficient derivative of this strain (W83ΔPPAD) to detect possible concentration-dependent differences. We then visualized the fate of the OMVs by fluorescence microscopy using anti-P. gingivalis antibodies. As documented in Fig. 2; Fig. S1A, S2, and S3A; and the Z-stack Videos S1 and S2, irrespective of the amount of OMVs applied, the OMVs were localized to the neutrophil cell surface. In particular, individual OMV-specific fluorescent signals were observed on the neutrophil surface when using 1 μg of OMVs (Fig. S1A). Subsequently, we incubated neutrophils with 5 μg OMVs for 30, 90, and 150 min (Fig. S2A and B). Also at this higher concentration, we did not observe OMV internalization at any of the three time points, indicating that neutrophils did not internalize the P. gingivalis OMVs, even upon extended periods of incubation. Thus, contrary to our previous observations with P. gingivalis viable cells (4), which are readily phagocytosed by human neutrophils, OMVs position themselves only on the neutrophil surface without being internalized.

To determine whether the unprecedented adhesive and noninternalizing behavior of P. gingivalis OMVs is exclusive to the OMV-neutrophil interaction, we also challenged macrophages and A-253 submaxillary salivary gland epithelial cells with P. gingivalis wild-type and PPAD-deficient OMVs. In contrast to neutrophils, the macrophages and A-253 cells were able to internalize these OMVs (Fig. 2; Fig. S3B and C; Z-stack Videos S3 and S4). The latter observations are in agreement with previous reports that P. gingivalis OMVs can be internalized by human THP-1 monocytes and THP1-derived macrophages (31) and by different human epithelial cells (32, 33). Further, polymerization of actin was observed in macrophages with internalized OMVs (Fig. 2). This implies that macrophage engulfment of OMVs is probably actin-mediated, as previously proposed for OMV entry into human host cells (3, 34). Since no significant differences in the association or internalization of PPAD-proficient or PPAD-deficient OMVs on/in neutrophils, macrophages and epithelial cells were observed, we conclude that protein citrullination plays no role in the differential OMV delivery.

To investigate whether gingipains have a role in the specific OMV exclusion by human neutrophils, we incubated the OMVs with the RgpA/B-specific inhibitor leupeptin and the Kgp-specific cathepsin B inhibitor II (35) for 30 min prior to their addition to the neutrophils. However, abundant OMV binding to the neutrophil surface and the absence of OMV internalization were still observed (Fig. S4A). Likewise, we tested whether plasma components might affect OMV adhesion or exclusion by the neutrophils by adding plasma to the culture medium of neutrophils prior to OMV addition. Also in this case, the OMV binding and exclusion by neutrophils was not altered (Fig. S4B). Together, these observations show that neither PPAD nor gingipains or plasma components have an impact on the specific association of OMVs with neutrophils and the lack of OMV internalization by these professional phagocytes.

### Neutrophil activation by OMVs.

**FIG 3:**
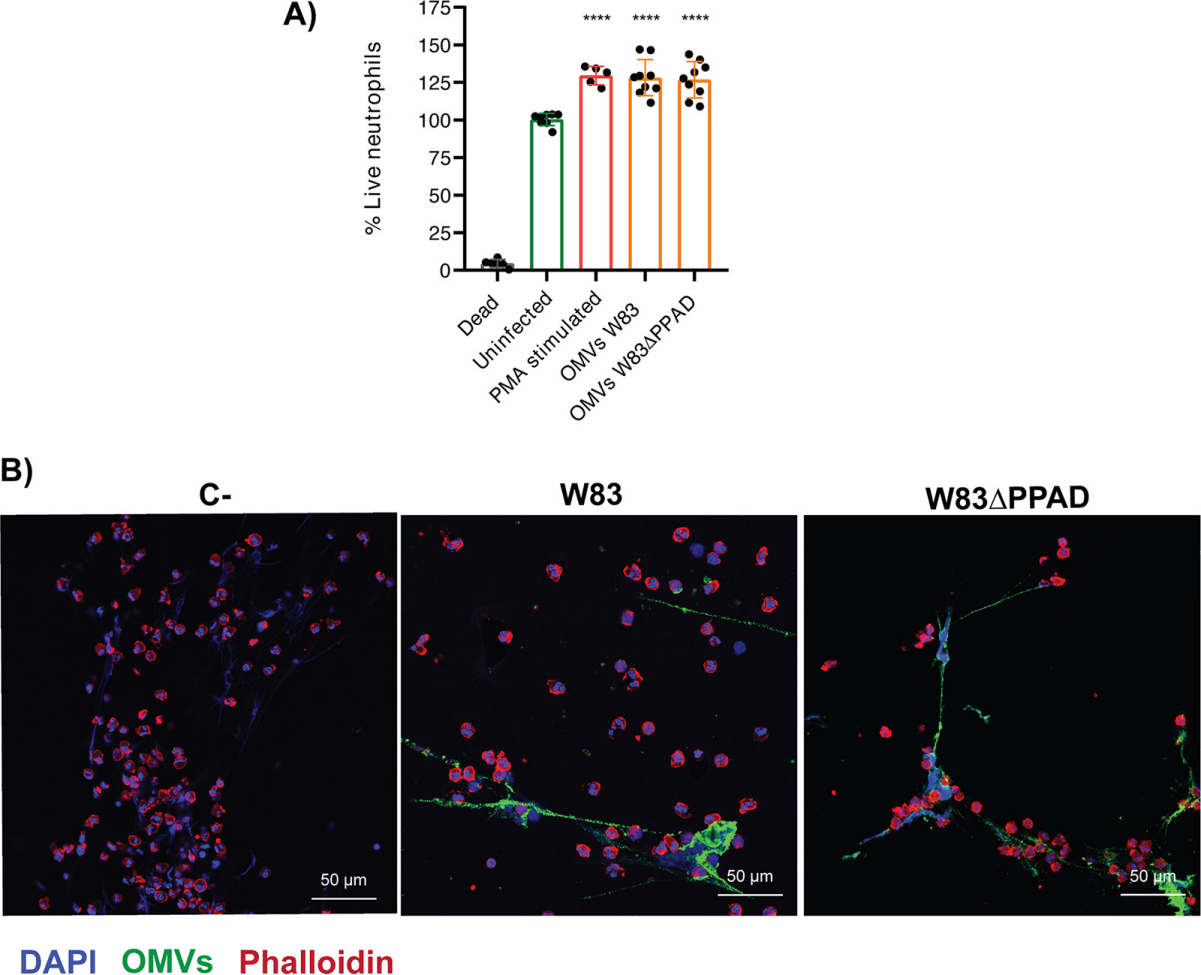
OMVs of P. gingivalis activate neutrophils without affecting their viability. (A) Percentage of live neutrophils as determined by MTT reduction assays upon challenge with 5 μg OMVs of the P. gingivalis W83 or W83ΔPPAD strains, relative to uninfected neutrophils. Alternatively, the neutrophils were activated with 20 nM phorbol myristate acetate (PMA). A percentage of live neutrophils greater than 100% was considered the result of neutrophil activation. To obtain a dead-cell control, neutrophils were treated with 0.1% SDS prior to addition of MTT. The data are presented as individual values, means, and standard deviation (SD) of three independent biological replicates with three technical replicates per biological replicate. Note that one biological replicate for the PMA-treated neutrophils was missed. Accordingly, statistical significance of the measurements was assessed with an ordinary one-way analysis of variance (ANOVA) followed by a multiple-comparison test to the control group; ****, *P* ≤ 0.0001. Furthermore, due to neutrophil stimulation by the treatment with OMVs or PMA, metabolic activity as reflected by the MTT activity was enhanced, resulting in live cell percentages of more than 100% relative to the control. (B) Representative confocal microscopy images of neutrophils and NETs with trapped OMVs of P. gingivalis strains W83 (middle image) or W83ΔPPAD (right image). OMVs were effectively trapped by the elongated NET structures observed by DAPI staining of the extracellular neutrophil DNA (blue). DAPI was also used to stain the nuclei of neutrophils that did not undergo NETosis (blue), and phalloidin-TRITC was used to stain actin (red). Unchallenged neutrophils used as a control (C-) are shown in the image on the left. Additionally, OMVs were labeled with specific antibodies (green) as described for Fig. 2. Bars mark 50 μm. The individual images used to create the overlays are presented in Fig. S5. NETs, neutrophil extracellular traps.

Next, we verified whether incubation with P. gingivalis OMVs would affect the viability of neutrophils, by measuring 3-[4,5-dimethylthiazol-2-yl]-2,5-diphenyl tetrazolium bromide (MTT) reduction, which results in the formation of readily detectable formazan crystals. Interestingly, we observed that the presence of OMVs did not negatively affect neutrophil viability as reflected by MTT reduction (Fig. 3A). On the contrary, elevated MTT reduction activity was observed in OMV-treated neutrophils, which reached similar levels as observed for phorbol myristate acetate (PMA)-stimulated neutrophils. Previous studies have shown that PMA-stimulated neutrophils display elevated MTT reduction (36), and we therefore conclude that the added OMVs resulted in neutrophil activation. Since activated neutrophils may produce NETs, we verified whether the addition of OMVs would provoke NETosis. This was not the case, but we did observe that the P. gingivalis OMVs were effectively captured in NETs, which can be spontaneously formed due to neutrophil activation upon isolation and handling of neutrophils (37, 38) (Fig. 3B; Fig. S1B).

### OMV-bound gingipains degrade human myeloperoxidase and LL-37 in neutrophil supernatants.

**FIG 4:**
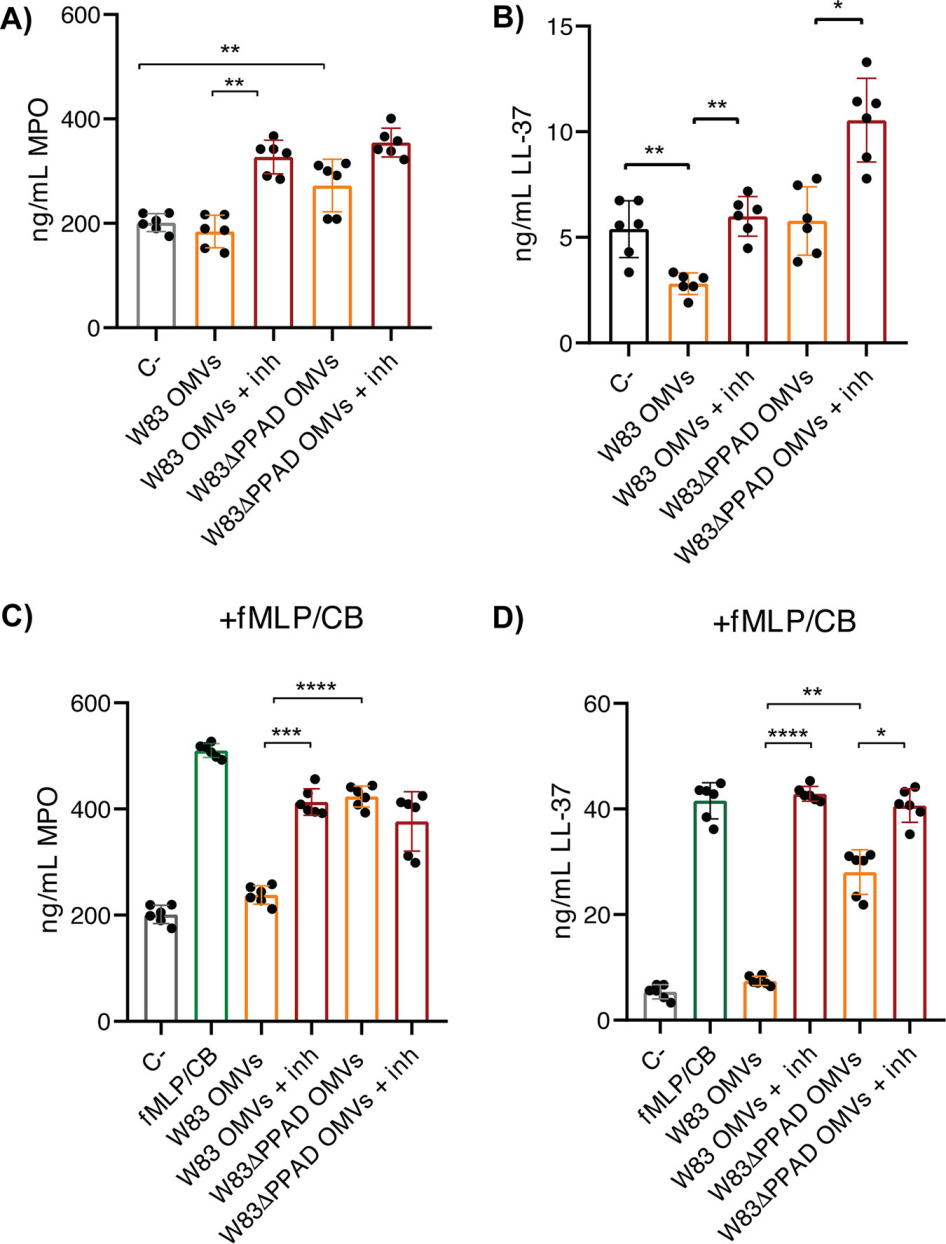
Degradation of human MPO and LL-37 released by neutrophils *via* OMV-bound gingipains. (A to D) Secretion of MPO (A) and LL-37 (B) by OMV-stimulated neutrophils in the absence of induced degranulation or secretion of MPO (C) and LL-37 (D) in the presence of degranulation induced with fMLP and cytochalasin B (CB) was determined by ELISA. A total of 5 μg of OMVs of P. gingivalis W83 or W83ΔPPAD were used for each condition. Gingipain-specific inhibitors were added (+inh) to the culture medium to assess the effects of OMV-bound gingipains on the degradation of MPO and LL-37. The concentrations of secreted MPO and LL-37 (ng/mL) were determined by ELISA of neutrophil supernatants. The data are presented as individual values, means, and SD of three independent biological replicates with two technical replicates per biological replicate. Statistical significance was assessed with two-tailed unpaired Student’s *t* tests. *, *P* ≤ 0.05; **, *P* ≤ 0.01; ***, *P* ≤ 0.001; ****, *P* ≤ 0.0001. fMLP, N-formyl-L-methionyl-L-leucyl-phenylalanine.

In view of the OMV-dependent neutrophil activation, we investigated whether this would lead to degranulation. To this end, we analyzed the supernatant of OMV-activated neutrophils to detect degranulation-derived antimicrobial proteins, especially MPO and LL-37 by enzyme-linked immunosorbent assay (ELISA). As shown in Fig. 4A and B, the presence of OMVs from the wild-type strain W83 did not affect the level of MPO, whereas a reduced level of LL-37 was detected. Since this was indicative of LL-37 proteolysis, we performed the same experiment in the presence of leupeptin and cathepsin B inhibitor II. Indeed, this led to an increased level of LL-37 that matched the control (Fig. 4B). Interestingly, the detected MPO level was even higher than in the control when gingipain inhibitors were present, showing that the OMVs had induced neutrophil degranulation and that gingipains catalyze the degradation of granule-derived MPO and LL-37 (Fig. 4A and B). This pattern of MPO or LL-37 degradation by OMV-borne gingipains was even more pronounced when degranulation was induced with *N*-formyl-Met-Leu-Phe (fMLP; Sigma-Aldrich) and cytochalasin B (Fig. 4C and D). Under these conditions, PPAD was shown to be a relevant factor in MPO and LL-37 degradation, because PPAD-deficient OMVs degraded these two granule-derived proteins to lesser extents, even in the absence of gingipain inhibitors. This is consistent with a lowered gingipain activity in the absence of PPAD (4, 39). Of note, in the fMLP- and cytochalasin B-induced degranulation conditions, the gingipain inhibitors were unable to rescue all of the MPO released by the neutrophils in the presence of OMVs (Fig. 4C). In contrast, the OMV-dependent degradation of LL-37 was fully prevented by the gingipain-specific inhibitors, verifying the complete gingipain inhibition. These findings show that as-yet-unidentified proteases participate in the degradation of released MPO.

**FIG 5:**
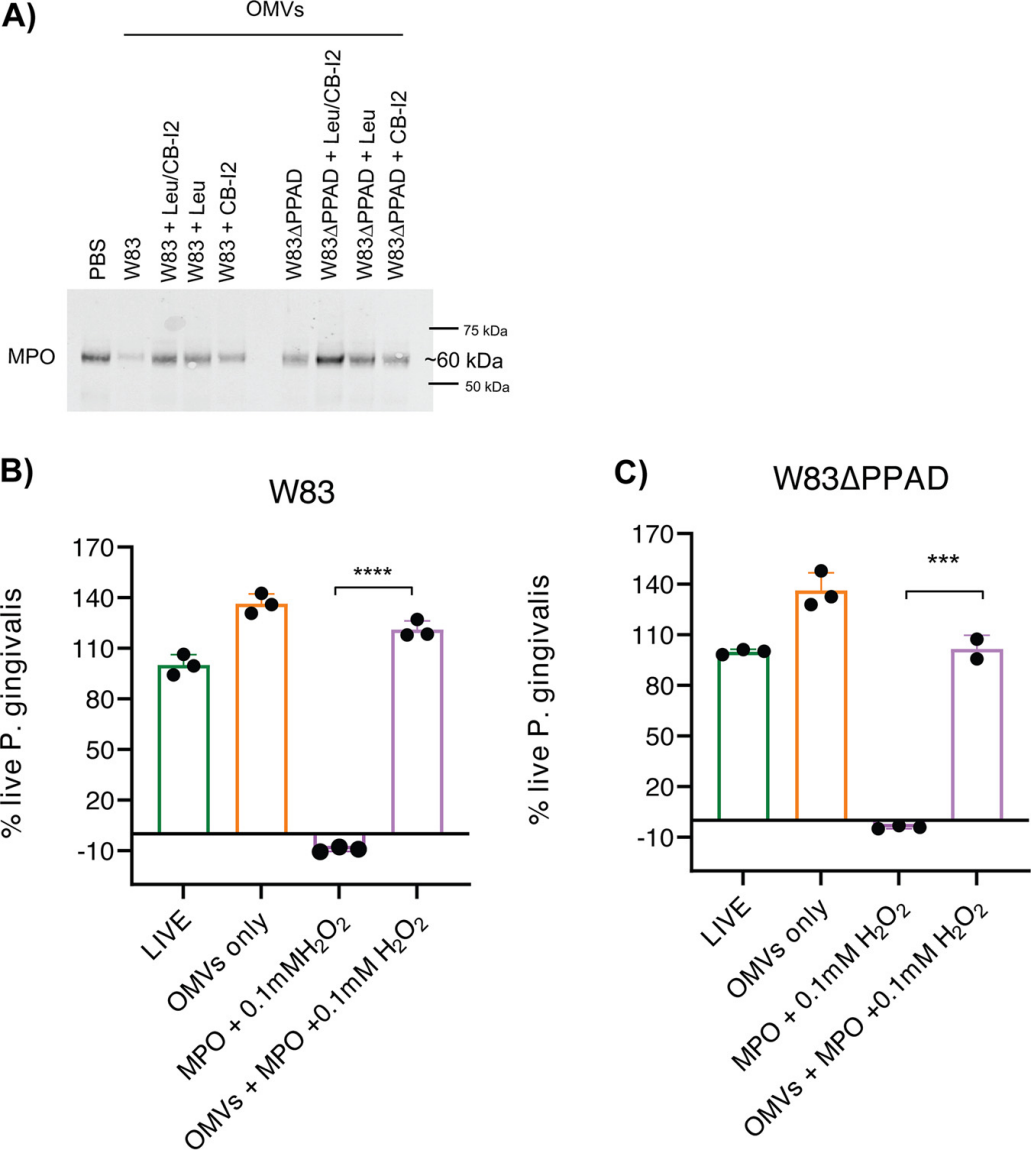
OMV-borne gingipains prevent myeloperoxidase-mediated killing of P. gingivalis. (A) Visualization of OMV-mediated and gingipain-specific MPO degradation by Western blotting. Equal protein amounts were loaded in each lane: 200 ng of MPO were incubated with PBS or 1 μg of OMVs derived from the P. gingivalis W83 or W83ΔPPAD strains. Gingipain activity was inhibited with Leupeptin (Leu) and/or cathepsin B inhibitor II (CB-I2). (B, C) Percentages of live P. gingivalis strains W83 (B) and W83ΔPPAD (C), as determined with LIVE/DEAD BacLight bacterial viability assay, upon challenges with MPO and H_2_O_2_ with or without OMV pretreatment. The numbers are relative to the untreated (100% live cells; LIVE) control and the killed P. gingivalis (0% live cells) control. Note that the addition of OMVs led to an apparently increased viability, probably due to SYTO 9 binding by the OMVs. On the other hand, negative viability values were obtained when the bacteria were incubated with MPO and H_2_O_2_, which could be attributed to effects of the generated reactive oxygen species (ROS) on the dyes applied for the LIVE/DEAD staining. The data are presented as individual values, means, and SD of three independent biological replicates with two technical replicates per biological replicate. Statistical significance was assessed with two-tailed unpaired Student’s *t* tests. ***, *P* ≤ 0.001; ****, *P* ≤ 0.0001. PBS, phosphate-buffered saline.

To validate the ELISA results, a Western blotting was performed with a granule-derived MPO preparation. This analysis underscored the difference in the MPO-degrading capacity of PPAD-proficient and -deficient OMVs (Fig. 5A). Of note, no complete degradation of MPO was observed in the presence of OMVs, which implies that a subfraction of granule-derived MPO was inaccessible to the OMV-associated gingipains. Furthermore, leupeptin and cathepsin B inhibitor II prevented MPO degradation, especially when applied in combination (Fig. 5A). This shows that both OMV-associated RgpA/B and Kgp are able to degrade human MPO, which is consistent with the fact that the 745-residue MPO protein contains 66 arginine and 22 lysine residues.

Lastly, to determine whether the observed effect of PPAD deficiency on MPO degradation was related to the previously described citrullination of gingipains (5, 40) or the possible citrullination of MPO by PPAD, we performed a mass spectrometric analysis of the MPO preparation incubated with or without recombinant PPAD. However, merely one potential citrullination event on arginine 569 of the MPO protein was detectable in one of three biological replicates that were analyzed (Fig. S6C; Table S1). This shows that the influence of PPAD on MPO degradation was most likely indirect.

### Gingipains inhibit MPO-mediated killing of P. gingivalis.

A key question to be answered was whether there is a benefit for P. gingivalis in remotely targeting neutrophils with OMVs. Therefore, we examined whether the observed OMV-dependent MPO degradation would affect the antibacterial activity of this hypochlorous acid-generating peroxidase. Accordingly, we assessed the viability of P. gingivalis in the presence of purified MPO and hydrogen peroxide. The results presented in Fig. 5B and C demonstrate that P. gingivalis is killed in the presence of MPO and hydrogen peroxide but that this killing activity is effectively prevented by the addition of OMVs. Importantly, at a hydrogen peroxide concentration of 0.005 mM, which does not kill P. gingivalis by itself, the presence of MPO led to a complete elimination of all bacteria in the assay, whereas MPO alone had no bactericidal activity (Fig. S6A and B). Together, these findings show that long-distance targeting of neutrophils with OMVs is an effective survival strategy of P. gingivalis in an inflamed tissue full of neutrophils.

### OMV exclusion by neutrophils is conserved.

**FIG 6:**
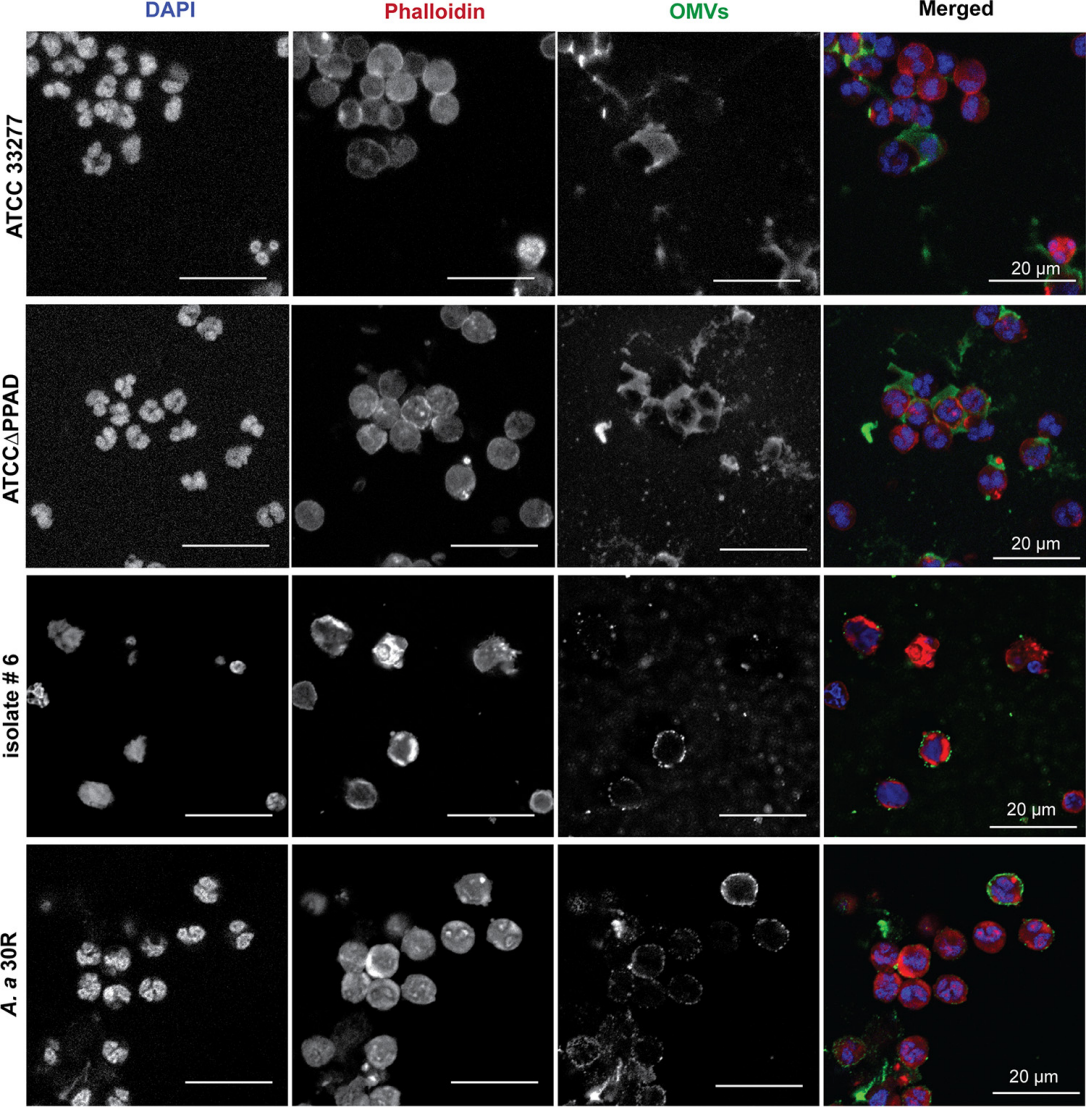
OMVs from other P. gingivalis strains and from A. actinomycetemcomitans escape neutrophil internalization. Shown are representative confocal fluorescence microscopy images of neutrophils challenged with OMVs from P. gingivalis strains ATCC 33277 and ATCCΔPPAD, the clinical isolate P. gingivalis isolate 6, and A. actinomycetemcomitans 30R. A total of 5 μg of OMVs were used for each experiment. DAPI was used to stain the neutrophils’ nuclei (blue), and phalloidin-TRITC was used to stain actin (red). OMVs were labeled with P. gingivalis- or A. actinomycetemcomitans-specific polyclonal rabbit antibodies and secondary goat anti-rabbit antibodies labeled with Alexa Fluor 488 (green). Bars in the panels with the merged images mark 20 μm. All tested OMVs bind to the neutrophil surface without being internalized.

**FIG 7:**
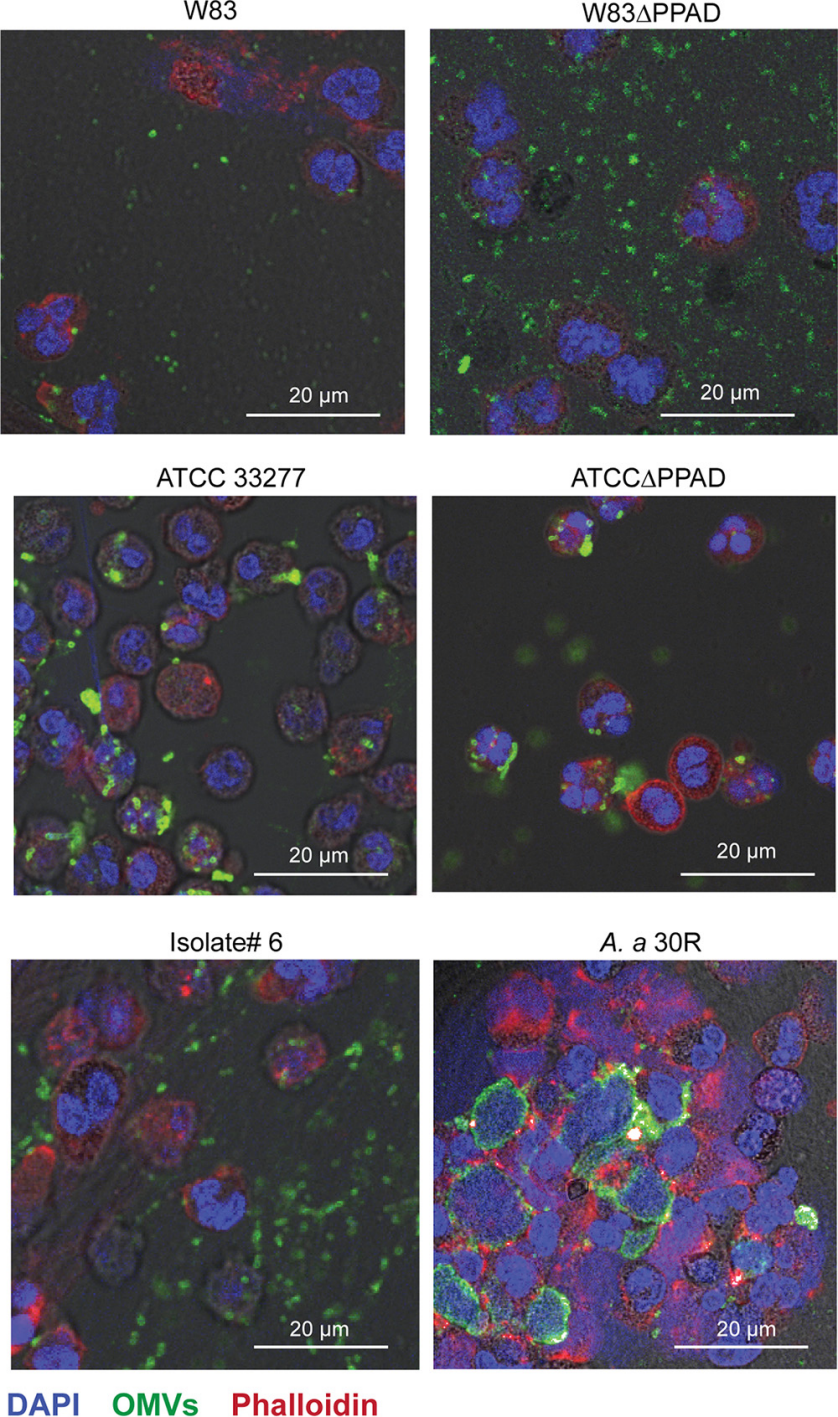
Interaction of P. gingivalis and A. actinomycetemcomitans with human neutrophils. Shown are representative confocal microscopy images of neutrophils challenged with P. gingivalis strains W83, W83ΔPPAD, ATCC 33277, or ATCCΔPPAD; the clinical isolate P. gingivalis isolate 6; or A. actinomycetemcomitans 30R. DAPI was used to stain the neutrophils’ nuclei (blue) and phalloidin-TRITC was used to stain actin (red). Bacterial cells were identified with P. gingivalis- or A. actinomycetemcomitans-specific polyclonal rabbit antibodies and secondary goat anti-rabbit antibodies labeled with Alexa Fluor 488 (green). Bars mark 20 μm. Note that P. gingivalis bacteria from strains W83 or W83ΔPPAD bind relatively poorly to neutrophils, whereas P. gingivalis bacteria from strains ATCC 33277 and ATCCΔPPAD and isolate 6 are more effectively internalized by the neutrophils. A. actinomycetemcomitans induced neutrophil lysis or NETosis as evidenced by the extracellular DAPI staining. The separate microscopy images used to create the overlays are presented in Fig. S7.

To verify whether OMV exclusion is conserved among P. gingivalis strains, we assessed the fate of PPAD-proficient and -deficient OMVs from the highly fimbriated strain ATCC 32277 and OMVs of clinical P. gingivalis isolate 6 (41) upon incubation with human neutrophils. Of note, the presence of fimbrial proteins in OMVs was previously shown to significantly increase OMV internalization by gingival fibroblasts and oral keratinocytes (42). Nonetheless, OMVs of the ATCC 33277 strain were still excluded by the neutrophils, as was the case for OMVs from clinical P. gingivalis isolate 6 (Fig. 6). In contrast, the living bacteria were bound and internalized by the neutrophils. This was particularly evident for the ATCC 33277 strain, which was internalized at a high rate irrespective of PPAD proficiency (Fig. 7). As previously demonstrated (4), PPAD-proficient and -deficient bacteria of the W83 strain were internalized, but to a much lower extent than observed for the ATCC 33277 strain (Fig. 7), and the same was true for bacteria of the PPAD-proficient isolate 6 (Fig. 7). These observations raised the question of whether OMV exclusion by neutrophils is conserved among different P. gingivalis lineages and also whether this OMV exclusion can also be observed for other major oral pathogens that need to withstand the neutrophil. We therefore isolated OMVs from the major fimbriated oral pathogen A. actinomycetemcomitans (isolate 30R) (43) and incubated them with human neutrophils. Indeed, the A. actinomycetemcomitans OMVs effectively coated the neutrophil surface but without being internalized and without detectably affecting the neutrophil integrity (Fig. 6). In contrast, some living A. actinomycetemcomitans bacteria were internalized (Fig. 7), but since the living bacteria triggered NETosis, many of them were captured in NETs. Altogether, these observations show that OMV exclusion by neutrophils is a conserved feature for the two major oral pathogens P. gingivalis and A. actinomycetemcomitans, irrespective of the fate of the OMV-producing bacteria upon close encounters with neutrophils.

## DISCUSSION

Colonization of the gingival crevice by the oral keystone pathogen P. gingivalis is a serious provocation of the human innate immune defenses (21, 24, 25, 28). Consequently, this Gram-negative bacterium has to withstand waves of neutrophils that migrate into the inflamed gingival tissue. It is therefore advantageous for P. gingivalis bacteria to fight these phagocytes from a distance. Here, we show for the first time that P. gingivalis can achieve this life-saving objective by projecting its OMVs against the wall of neutrophils, thereby defusing the antimicrobial activities of the granule-derived proteins MPO and LL-37. This unprecedented observation adds to the growing list of different functions that have been attributed to Gram-negative bacterial OMVs. In particular, previous research has implicated such OVMs in bacterial pathogenesis (44); extracellular compartmentalization to protect OMV cargo proteins against proteolysis (45); the acquisition of iron and zinc (46); the disposal of detrimental compounds like peptidoglycan fragments, mislocalized lipopolysaccharide (LPS), outer membrane protein aggregates, and antimicrobial peptides (46, 47); and the promotion of interbacterial interactions and biofilm formation (48, 49). Furthermore, OMVs were shown to serve as decoys for antibodies, antimicrobials, and bacteriophages (50). Lastly, despite their role in pathogenesis, OMVs can potentially elicit protective immune responses in the mammalian host, which is critically underscored by the current use of OMV-based vaccines against Neisseria meningitidis serogroup B (51).

Remarkably, the OMVs of P. gingivalis coat the neutrophils without being internalized. Instead, the strategically positioned OMVs on the neutrophil surface activate the neutrophil to degranulate, as we demonstrate here for the highly virulent P. gingivalis strain W83. Subsequently, the gingipain cargo of the OMVs can effectively degrade bactericidal granule-derived proteins and peptides, such as MPO and LL-37. Our observations show that even upon release of larger quantities of these antimicrobial compounds through degranulation, the gingipain-containing OMVs will still efficiently degrade MPO and LL-37. This is fully in line with the previously reported degradation of other human-derived proteins by gingipains (27, 52–54). Notably, we show that the proteolytic capacity of gingipains is higher in PPAD-proficient OMVs than in PPAD-deficient OMVs, consistent with PPAD-mediated enhancement of gingipain activity by removal of potential sites for self-cleavage through citrullination (39, 55). It thus seems that the OMVs of P. gingivalis induce inflammatory responses, even without affecting the neutrophil’s viability. Importantly, the successful deployment of OMVs in what seems to be a neutrophil-deceptive strategy for colonizing gingival crevices is conserved in P. gingivalis. To date, neutrophils are the first and only human cells that bind but cannot internalize OMVs, despite the presence of fimbrial proteins that otherwise promote internalization as demonstrated for the ATCC 33277 strain (42, 56). Intriguingly, we now show that at least one other periodontitis-associated bacterium, A. actinomycetemcomitans, also produces OMVs that coat but do not enter the neutrophil. At present, it is not known whether neutrophil coating by OMVs is a receptor-mediated phenomenon or whether proteins are involved in this process. However, considering the finding that the intact P. gingivalis ATCC 33277 bacteria are effectively internalized by neutrophils, while the OMVs of different strains and bacteria all show neutrophil coating, we conclude that OMV exclusion relies on specific biochemical or biophysical features. These features differ from those of the viable bacteria, which show a differential behavior when in close contact with a neutrophil. It will be an important challenge for our future studies to determine the molecular basis for the observed OMV exclusion by human neutrophils. Another important question that remains to be answered is how many OMVs are secreted by P. gingivalis *in vivo*. Clearly, the growth conditions in the gingival crevice of a patient with periodontitis will differ substantially from the presently applied *in vitro* culture conditions, and this may have consequences for the amounts of OMVs that are produced by P. gingivalis.

## MATERIALS AND METHODS

### Bacterial culture.

P. gingivalis strains W83 and ATCC 33277, their respective PPAD-deletion mutants (W83ΔPPAD and ATCC ΔPPAD), and clinical isolate 6 (originally designated 512915) were cultured as previously described (40). Briefly, these strains were streaked on blood agar no. 2 (BA2) plates, and growth was allowed for 5 days under anaerobic conditions at 37°C. Subsequently, liquid cultures in brain-heart infusion (BHI; Oxoid) supplemented with 5% l-cysteine, 5 mg/liter hemin, and 1 mg/liter menadione were incubated overnight at 37°C under anaerobic conditions until the early stationary phase was reached as determined by optical density measurements at a wavelength of 600 nm (OD_600_).

The clinical A. actinomycetemcomitans isolate 30R was grown on BHI agar plates supplemented with 5% l-cysteine, 5 mg/liter hemin, and 1 mg/liter menadione at 37°C and in 5% CO_2_ (43). After 3 days, A. actinomycetemcomitans colonies were picked from BHI agar plates and cultured in BHI broth overnight until it reached a midexponential phase at an OD_600_ of ~0.5.

### P. gingivalis and A. actinomycetemcomitans OMV isolation, purification, and quantification.

For P. gingivalis, OMVs were harvested from early stationary-phase cultures (OD_600_ = ~2.5 to 3.0). To remove all bacterial cells, the culture samples were centrifuged at 8,000 × *g* for 15 min at 4°C, as previously described (57, 58). The OMV-containing supernatant was carefully collected, filtered through a 0.22-μm filter (GE Healthcare Life Sciences), and ultracentrifuged overnight at 100,000 × *g* at 4°C, in an Optima XL-80K ultracentrifuge using a SW32-TI rotor. The OMV pellet was washed once with phosphate-buffered saline (PBS) before proceeding with the OMV purification using an iodixanol (Optiprep) density gradient. OMVs were solubilized in 50% iodixanol (i.e., the highest concentration used) in 0.85% (wt/vol) NaCl, 60 mM HEPES-NaOH (pH 7.4; buffer A). Subsequent layers of Optiprep (40, 30, 20, and 10%) were prepared in buffer B, which consisted of 0.85% (wt/vol) NaCl, 10 mM HEPES-NaOH (pH 7.4). Ultracentrifugation of the gradient was performed at 100,000 × *g* at 4°C using an Optima Max-XP ultracentrifuge (Beckman Coulter) and an MLA-80 rotor. As previously shown (59), the OMVs accumulated at 20 to 30% Optiprep. The respective fractions were carefully collected, pooled, diluted with buffer B, and ultracentrifuged overnight at 100,000 × *g* at 4°C. The OMV pellet was washed with PBS to remove all the Optiprep, and ultracentrifugation was performed again for 2 h. The OMV pellet was resuspended in PBS, and the OMV solution was sterilized using a 0.22-μm filter (GE Healthcare Life Sciences) before freezing in aliquots at –80°C. Quantification was performed by determining the OMV protein concentration using the Pierce bicinchoninic acid (BCA) protein assay kit (Thermo Fisher Scientific). To solubilize OMVs, they were first treated with 2% SDS before performing the protein quantification (60). On average, we obtained per 200 mL of culture about 1 to 2 mL of OMVs at a protein concentration of about 600 μg/mL. This means that per mL of culture, we obtained between 3 and 6 μg of OMVs. A. actinomycetemcomitans OMVs were isolated following the same protocol as for P. gingivalis, with the exception that the OMVs were harvested after 24 h culturing.

### Cell lines and primary cell cultures.

**(i) A253 submaxillar salivary gland epithelial cells.** A253 cells were maintained in Dulbecco’s modified Eagle’s medium (DMEM, Sigma-Aldrich) supplemented with 10% fetal bovine serum (FBS; Sigma-Aldrich).

**(ii) Human neutrophils.** Neutrophils were isolated from fresh blood of healthy female donors (aged 29 to 31 years old), who had been medically examined beforehand. The protocol for neutrophil isolation was performed using the Lymphoprep buffer as described previously (4). The peripheral blood mononuclear cells (PBMCs) were kept separately when the primary macrophages were used (see the following section). To lyse the erythrocytes that were present in the neutrophil-containing layer, the RBC lysis buffer (BioLegend) was used at 1×. Neutrophils were resuspended in RPMI medium (Gibco) supplemented with 2 mM l-glutamine (Thermo Fisher Scientific) and 10% autologous donor plasma (termed as neutrophil culture medium).

**(iii) Human primary macrophages.** Human PBMCs were isolated as described above. The monocytes were differentiated into macrophages *in vitro* using treatment with macrophage colony-stimulating factor (M-CSF; Gibco) recombinant human protein. Briefly, PBMCs were resuspended in RPMI medium (Gibco) supplemented 2 mM l-glutamine (Thermo Fisher Scientific) and 10% autologous donor plasma. A total of 2.5 × 10^5^ monocytes were seeded on sterile no. 1.5 round glass coverslips (Thermo Fisher Scientific) in 24-well plates and were allowed to adhere for 24 h in 5% CO_2_ at 37°C. After this time, the medium was replaced, and M-CSF was added at a final concentration of 50 ng/mL. The cells were incubated for a total of 6 days, during which the medium was replaced every 2 days with fresh supplemented RPMI also containing M-CSF. The cells were regularly inspected microscopically to check the confluence and morphology changes (macrophages should possess more granules, are bigger in size, and are a bit elongated). All cell counting was performed using a LUNA-II cell counter (Logos biosystems) with trypan blue staining to determine the percentage of live cells.

### Invasion experiments using P. gingivalis OMVs.

For OMV invasion experiments with A253 cells, a total of 1.0 × 10^5^ cells/well was used and seeded on sterile coverslips. After 2 days of incubation in 5% CO_2_ at 37°C, the cells reached the desired confluence before OMV addition (5 μg). The cells were cultured for 24 h in 5% CO_2_ at 37°C. For primary macrophage invasion experiments, 1 μg of P. gingivalis OMVs was used, and the macrophages were cultured for 90 min in 5% CO_2_ at 37°C.

For neutrophil invasion experiments, 5 × 10^5^ neutrophils were seeded in a 24-well plate in 500 μL of neutrophil culture medium. For immunostainings, the coverslips were first washed with 1 M HCl to increase neutrophil attachment and avoid neutrophil activation (61). Subsequently, the coverslips were sterilized and placed in a 24-well plate prior to neutrophil seeding. The neutrophils were incubated for 30 min at 37°C with 5% CO_2_ to allow cell attachment to the plates or coverslips. Incubation with P. gingivalis OMVs was performed for 90 min at 37°C. To determine the optimal OMV concentration for infection, initially different OMV amounts were applied (1 or 5 μg OMVs). A time course experiment was also performed using 5 μg of OMVs/well for 30, 90, and 180 min at 37°C, 5% CO_2_. For subsequent experiments, we used 5 μg of OMVs/well and an infection time of 90 min.

For some samples, the same experimental setup was used separately without the addition of human plasma to the neutrophil culture medium in order to assess a possible role of plasma components in the interaction of OMVs with the neutrophils. Similarly, to study the effect of gingipain activity in the OMV-neutrophil interactions, gingipains were inhibited with gingipain-specific inhibitors. For this, 100 μM Kgp inhibitor cathepsin B inhibitor II (Merck) and 100 μM Rgp inhibitor leupeptin (Sigma-Aldrich) were added during the neutrophil invasion experiments. All coverslips with cells were fixed with 4% paraformaldehyde (PFA) for 15 min at room temperature (RT) and stored at 4°C in PBS until use for immunostaining and confocal fluorescence microscopy.

### Neutrophil infection experiments with P. gingivalis and A. actinomycetemcomitans bacteria.

Neutrophil infection experiments were performed using the same conditions as described for the OMV challenge experiments. The multiplicity of infection (MOI) for all P. gingivalis strains used (W83, W83ΔPPAD, ATCC 33277, ATCCΔPPAD, and clinical isolate 6) was 100. For A. actinomycetemcomitans, an MOI of 25 was used. After 90 min of incubation with the neutrophils, the coverslips with cells were fixed as described above.

### Antibodies against whole-cell P. gingivalis or A. actinomycetemcomitans.

Polyclonal antibodies against whole P. gingivalis cells were raised at Eurogentec. To this end, P. gingivalis W83 and A. actinomycetemcomitans 30R were fixed with 1% PFA and washed five times with PBS prior to immunization of a rabbit following a protocol provided by the supplier. Selection of the rabbit to be immunized was performed based on the screening of preimmune sera without cross-reactivity toward P. gingivalis. Subsequently, the specificity of antibodies against P. gingivalis or A. actinomycetemcomitans and its OMVs was verified by Western blotting and immunostaining.

### Immunostaining.

Coverslips with fixed samples were first washed once with PBS. Following this step, neutrophil membranes were permeabilized using 0.5% Tween 20 for 15 min at RT. Subsequently, a blocking step was performed with 1% bovine serum albumin (BSA) for 1 h at RT. Incubation with primary rabbit polyclonal antibodies raised against whole P. gingivalis cells or whole A. actinomycetemcomitans was performed for 1 h at RT, which was followed by two washes with PBS. Subsequently, incubation with either Alexa Fluor 488, Alexa Fluor 647, or Alexa Fluor 555 goat anti-rabbit antibodies (Invitrogen) was performed for 30 min at RT in the dark. Neutrophil nuclei were stained at this step with 4′,6-diamidino-2-phenylindole (DAPI; Sigma-Aldrich). In some cases, actin was visualized using tetramethyl-rhodamine B isothiocyanate-phalloidin (TRITC-phalloidin; Sigma-Aldrich). After washing two times with PBS, the coverslips were mounted on polylysine slides (Thermo Fisher Scientific) using Mowiol 4-80 as the mounting medium (Sigma-Aldrich). All washing steps and incubations were done carefully to avoid detachment of cells, especially neutrophils, from the coverslips. The mounting medium was dried by overnight incubation at RT in the dark.

### Confocal fluorescence microscopy.

The images were recorded with a Leica SP8 confocal microscope (Leica Microsystems) and analyzed using the LAS X software. For selected images, three-dimensional reconstructions from Z-stacks of two-dimensional confocal microscopy images were made using the Imaris 7.6.5 software.

### Viability of OMV-challenged neutrophils.

The viability of neutrophils after challenge with P. gingivalis OMVs was assessed using the MTT assay. For this, 5 × 10^5^ neutrophils were seeded in each well of a 96-well plate and treated with 5 μg of OMVs for 90 min at 37°C with 5% CO_2_ in neutrophil culture medium. The total volume in each well after this step was 150 μL. Subsequently, 15 μL of 3.0 mg/mL MTT was added (final concentration, 0.27 mg/mL), and the plate was further incubated for 3 h. Carefully, 100 μL of the liquid were removed from the top of the wells, such that the formazan crystals formed at the bottom of the well were not disturbed. Then, 190 μL of DMSO were added to each well. Lastly, the crystals were dissolved by pipetting up and down the contents of each well until the solution was homogeneous. A dead-cell control was prepared by lysing the neutrophils with 0.1% SDS prior to addition of MTT. In parallel, neutrophils were activated with 20 nM PMA as a control for activated neutrophils. The optical density of each well was measured at 570 nm. For each sample, the average blank value (no cells) was subtracted from each average sample value, and the percentage of viable cells was calculated with the following equation: percentage viability = (average sample value/average of uninfected value) × 100. Five technical replicates of each condition were used.

### Induction of degranulation.

To study the proteolytic effect of OMV-bound gingipains on neutrophil-derived proteins, we used two experimental conditions. The first condition involved the addition of OMVs to unstimulated neutrophils. The second condition involved the addition of OMVs to neutrophils and subsequent induction of degranulation by treatment with 10 μM cytochalasin B (Sigma-Aldrich) at 37°C and 5% CO_2_ for 5 min, followed by 10 μM fMLP for 30 min. Subsequently, the neutrophil supernatant fractions were collected for enzyme-linked immunosorbent assays (ELISAs). Gingipains were inhibited with gingipain-specific inhibitors for each condition tested. For this, OMVs were incubated with 100 μM Kgp inhibitor cathepsin B inhibitor II (Merck) and/or 100 μM Rgp inhibitor leupeptin (Sigma-Aldrich) for 30 min at 37°C prior to addition to the neutrophils. Three replicates per sample were used.

### ELISA of MPO and LL-37.

After challenging neutrophils with OMVs, the supernatant fraction of each well was collected in a 1.5-mL tube. The tubes were then centrifuged at 400 × *g* for 5 min at 4°C to pellet any cells that had detached from the wells during the supernatant collection. Subsequently, the cell-free supernatant was transferred to a second 1.5-mL tube and frozen at –80°C until analysis. Collected culture supernatants were used to determine MPO and LL-37 concentrations using ELISAs according to the manufacturer’s instructions (HycultBiotek).

### Purification of myeloperoxidase.

Myeloperoxidase was purified from pooled buffy coats of five healthy neutrophil donors as described previously (62). The OD 428/280 ratio of the MPO preparation was measured with NanoDrop One (Isogen Lifescience) to assess the purity of MPO relative to the total protein content, and the MPO concentration was calculated using the molar extinction coefficient of 91,000 M^−1 ^cm^−1^ per heme. The MPO purity was ~50% with a final MPO concentration of 1.0 mg/mL.

### Analysis of MPO degradation by Western blotting.

To assess MPO degradation by OMV-bound gingipains, we incubated 200 ng of MPO with 1 μg of OMVs. Gingipain inhibitors were preincubated with OMVs for 30 min prior to addition to the MPO samples. After incubation, the proteins were precipitated with 10% trichloroacetic acid (TCA) as described previously (41). The protein pellet was resuspended in loading buffer consisting of lithium dodecyl sulfate (LDS) sample buffer (Life Technologies), sample reducing agent (Life Technologies), and Milli-Q water. The samples were then boiled for 10 min at 95°C before loading on 10% NuPAGE gels (Life Technologies). Subsequently, Western blotting analysis was performed using a semidry system to transfer proteins to a 0.45-μm nitrocellulose membrane (GE Healthcare Life Sciences). After transfer, the membrane was blocked overnight with 5% skim milk (Oxoid Limited). The next day, the membrane was washed once with PBS and incubated with a polyclonal rabbit anti-human MPO antibody (Dako, Glostrup, Denmark) at a dilution of 1:10,000 in PBS-Tween 20 (PBS-T) for 2 h at RT. Next, the membrane was washed three times with PBS-T and incubated with goat anti-rabbit IRDye700CW (Invitrogen) at a dilution of 1:10,000 in PBS-T for 45 min at RT. Afterwards, the membrane was washed with PBS-T three times and with PBS two times before scanning of the membrane using an Amersham Thyphoon RGB (GE Healthcare Life Sciences).

### Sample preparation for citrullination analysis in MPO and MPO + PPAD samples.

The composition of the purified MPO preparation and MPO citrullination by PPAD were analyzed by mass spectrometry (MS). For the latter purpose, recombinant PPAD was purified from Lactococcus lactis as described previously (41, 63). Subsequently, PPAD (1 μg) was incubated with MPO (2 μg) overnight at 37°C.

Purified MPO and MPO + PPAD samples were prepared according to Hughes et al. (64) applying the single-pot, solid-phase-enhanced sample-preparation (SP3) protocol. In brief, the samples were reduced with tris-(2-carboxyethyl)phosphine (TCEP; 5 mM for 45 min at 65°C) and alkylated with iodoacetamide (IAA; 10 mM for 20 min at room temperature in the dark). SP3 beads (2 μL) (hydrophobic: Sera-Mag Speedbeads carboxylate-modified particles [GE Healthcare]; hydrophilic: Speedbead magnetic carboxylate-modified particles [GE Healthcare]) were added to the samples. Subsequently, ethanol was added to a final concentration of 50% (vol/vol), and the samples were incubated in a thermomixer (for 5 min at 24°C at 900 rpm). The tubes were placed in a magnetic rack to collect the beads, and the supernatant was removed. The beads were washed twice with 80% (vol/vol) ethanol and air dried. Afterwards, the proteins were digested by adding 25 μL digestion buffer (50 mM triethylammonium bicarbonate [TEAB]) containing 100 ng of trypsin followed by 30 s sonication in a water bath to disaggregate the beads, and incubation at 37°C was continued for 18 h. The beads were removed by centrifugation (20,000 × *g*, 1 min) and by placing the tubes in a magnetic rack to transfer the supernatant to a glass vial. The supernatants were dried by vacuum centrifugation, and the peptides were reconstituted in 10 μL 0.1% acetic acid in water. Finally, the samples were analyzed by MS.

### Mass spectrometric analysis.

All MPO samples were analyzed by reversed-phase liquid chromatography (LC) electrospray ionization (ESI) MS/MS. Thus, an EASY-nLC 1000 (Thermo Fischer Scientific) was coupled to a QExactive mass spectrometer (Thermo Fischer Scientific). The peptides were loaded onto in-house self-packed fused silica columns (75 μm × 20 cm) containing reversed-phase C18 material (ReproSil-Pur 120-AQ 1.9 μm, Maisch GmbH). The peptides were eluted using a nonlinear binary gradient of 31 min from 2 to 99% solvent B (0.1% [vol/vol] acetic acid in acetonitrile) in solvent A (0.1% [vol/vol] acetic acid) at a constant flow rate of 300 nl/min. The full scan was recorded with a mass range from 300 to 1,650 *m*/*z* and a resolution of 70,000 at 200 *m*/*z*. The 10 most abundant ions were isolated with an isolation width of 3 Th and fragmented by higher-energy collisional dissociation (HCD) at a normalized collision energy (NCE) of 27. Fragment ion spectra were recorded with a resolution of 17,500 at 200 *m*/*z*. Ions with unassigned charge states as well as charge 1 and higher than 6 were excluded from fragmentation. Fragmented ions were excluded from fragmentation for 10 s. Lock mass correction was enabled.

### Database search and manual validation of citrullination sites.

For database searches, the spectra were searched using MaxQuant with the implemented Andromeda search algorithm (version 1.6.17.0, Max Planck Institute of Biochemistry) (65, 66) against the Human Reference Proteome (reviewed) downloaded from Uniprot (September 28, 2020). Parameters for precursor mass deviation were set to 4.5 and 20 ppm for fragment mass tolerance. A false discovery rate (FDR) of 0.01 on protein, peptide, and spectral levels was applied, as well as a minimum peptide length of seven amino acids and two unique peptides per protein. Full tryptic specificity with a maximum of two missed cleavage sites was applied. Variable modifications were oxidation on methionine (M) and N-terminal acetylation (Protein N-term), citrullination on arginine (R), and deamidation on asparagine and glutamine (NQ). Furthermore, MaxQuant’s generic contamination list was included.

Classical database search algorithms still overestimate the number of potential citrullinated peptides and citrullination sites. Therefore, a manual validation according to Lee et al. (67) of all spectra, including potential citrullination, was indispensable. Accordingly, the spectra were inspected for the presence of diagnostic ions with the neutral loss of isocyanic acid, the presence of the immonium ion at 130.097 *m*/*z*, and the incomplete fragment ion series without N/Q ambiguity.

### LIVE/DEAD BacLight bacterial viability assay to assess P. gingivalis viability.

The bactericidal effect of MPO was assessed using the LIVE/DEAD BacLight bacterial viability assay (Thermo Fisher Scientific) and a BioTek Synergy 2 microtiter plate fluorescence reader. For this, P. gingivalis W83 and W83ΔPPAD cultures were grown overnight as described above, and 2 × 10^7^ CFU/mL were used to seed individual wells of a 96-well plate. Living and killed bacterial control suspensions were prepared according to the manufacturer’s instructions. The purified MPO preparation described above was used at a concentration of 5 nM MPO/well. Furthermore, different concentrations of H_2_O_2_ were tested (0.6, 0.1, and 0.005 nM) in combination with MPO. Upon addition of MPO, H_2_O_2_, or MPO+H_2_O_2_ to the living bacteria, the plate was incubated for 90 min at 37°C. A stock solution of propidium iodide and SYTO 9 was prepared according to the manufacturer’s instructions and added to the samples, and the bacteria were subsequently incubated at RT in the dark for 15 min. Fluorescence was measured using an excitation wavelength of 485 nm and emission wavelengths of 530 and 620 nm.

Preincubation of MPO and OMVs was also performed to assess whether MPO degradation hampered the killing of bacterial cells. For this, 3.5 μg of OMVs were incubated with 5 nM MPO for 90 min at 37°C before the addition of H_2_O_2_ and subsequent incubation with the bacterial cells. A well with only OMVs was used to assess the change in fluorescence that might be due to nucleic acids present in OMVs. This value was subtracted from all the OMV-containing samples. Live/dead cell ratios were calculated, and the percentage of living bacteria was calculated in relation to the wells with untreated living bacteria (100% live) and dead cells (0%). Three technical replicate measurements were performed for each condition.

### Statistical analyses.

The data were analyzed using GraphPad Prism version 8.0 for Windows (GraphPad Software). Statistical significance was determined using a paired two-tailed Student’s *t* test or a one-way analysis of variance (ANOVA) followed by a multiple-comparison test. A *P* value of ≤0.05 was considered significant. All experiments were replicated at least twice.

### Medical ethical approval.

Blood donations from healthy volunteers were collected with approval of the medical ethical committee of the University Medical Center Groningen (UMCG; approval no. Metc2012-375) upon written informed consent and in accordance with the Declaration of Helsinki guidelines.

### Data availability.

The raw data for all experiments and MaxQuant output tables were deposited to the ProteomeXchange Consortium via the PRIDE partner repository (68) (Pride Project accession no. PXD026704).
