# Supplementary material for: Coating and Corruption of Human Neutrophils by Bacterial Outer Membrane Vesicles
Source: Microbiol Spectr. 2022 Aug 24;10(5):e00753-22. doi: 10.1128/spectrum.00753-22 (PMC9602476; doi:10.1128/spectrum.00753-22)
Supplement: Supplemental file 1 — Fig. S1 to S7; captions to Table S1 and Videos S1 to S4. Download spectrum.00753-22-s0001.pdf, PDF file, 7.8 MB [file spectrum.00753-22-s0001.pdf]

Supplementary Figures

Coating and corruption of human neutrophils by bacterial outer membrane vesicles

Marines du Teil Espina[1], Yanyan Fu[1*], Demi van der Horst[1*], Claudia Hirschfeld[2], Marina López-Álvarez[1], Lianne M. Mulder[1], Costanza Gscheider[1], Anna Haider Rubio[1], Minke Huitema[3], Dörte Becher[2], Peter Heeringa[3], and Jan Maarten van Dijl[1#]

[1]Department of Medical Microbiology, University of Groningen, University Medical Center Groningen, Groningen, The Netherlands (m.du.teil.espina@umcg.nl; y.fu@umcg.nl; demivanderhorst@live.nl; m.lopez.alvarez@umcg.nl; lianne_mulder@hotmail.com; gscheider.c@gmail.com; a.haider.rubio@student.rug.nl; j.m.van.dijl01@umcg.nl)

[2]Institute for Microbiology, Ernst-Moritz-Arndt-University Greifswald, Greifswald, Germany (claudia.hirschfeld@uni-greifswald.de; dbecher@uni-greifswald.de)

[3]Department of Pathology and Medical Biology, University Medical Center Groningen, University of Groningen, Groningen, The Netherlands (m.g.huitema@umcg.nl; p.heeringa@umcg.nl)

*These authors contributed equally to this work

#Address correspondence to Jan Maarten van Dijl, j.m.van.dijl01@umcg.nl

Running title: Bacterial OMV exclusion by the neutrophil

Supplementary Figure S1

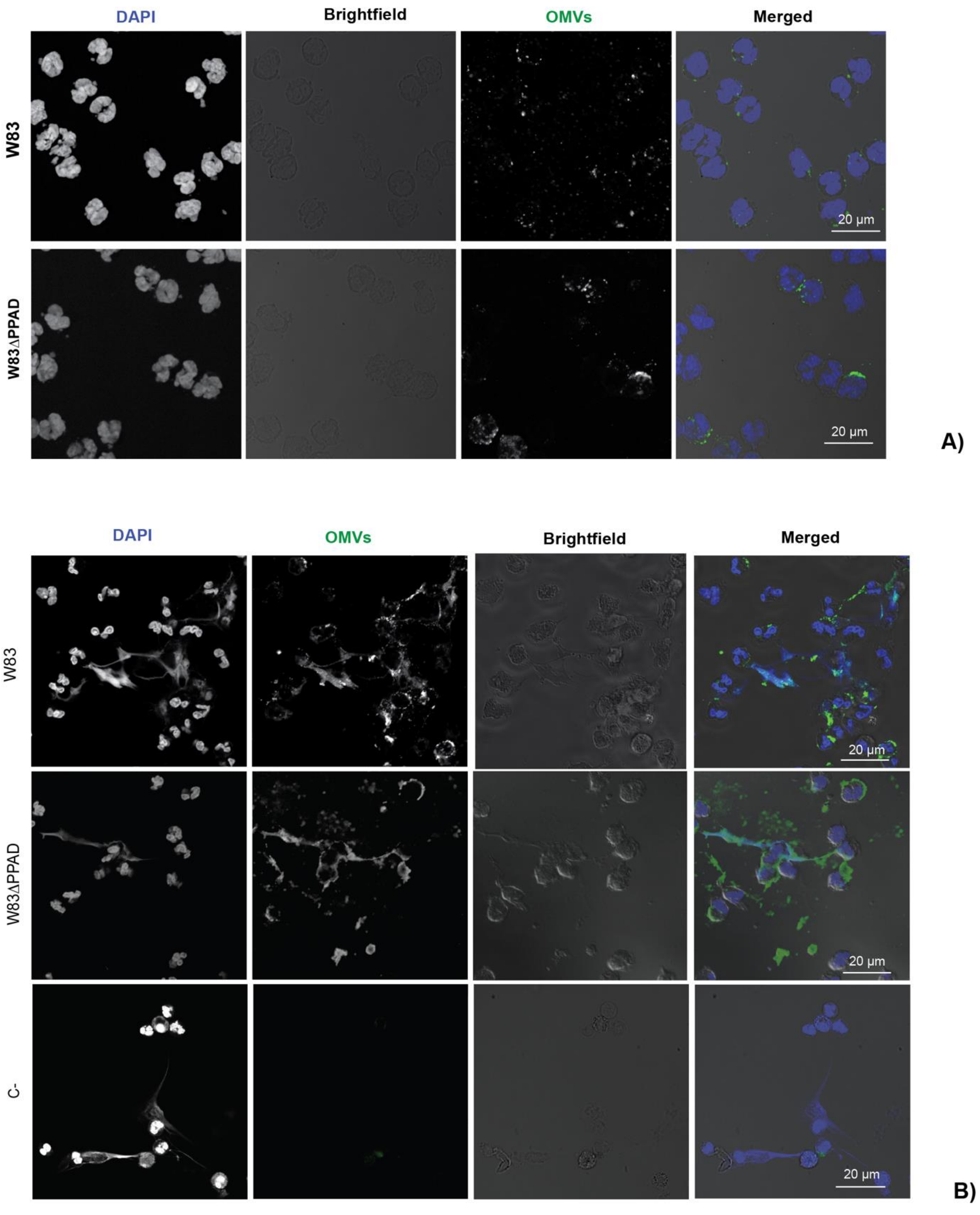

Supplementary Figure S1. OMVs (1 µg) of *P. gingivalis* coat the neutrophil and are trapped in NETs. (A and B) Confocal fluorescence microscopy images of human neutrophils challenged with OMVs from *P. gingivalis* W83 or W83ΔPPAD. (A) an amount of 1 µg of OMVs was used to detect single OMV-fluorescent signals. (B) NETs trapping OMVs of *P. gingivalis.* DAPI was used to stain the neutrophils' nuclei (blue) and OMVs were labelled with *P. gingivalis*-specific polyclonal rabbit antibodies and secondary goat-anti-rabbit antibodies labelled with AlexaFluor488 (green). Scale bars mark 20 µm.

Supplementary Figure S2

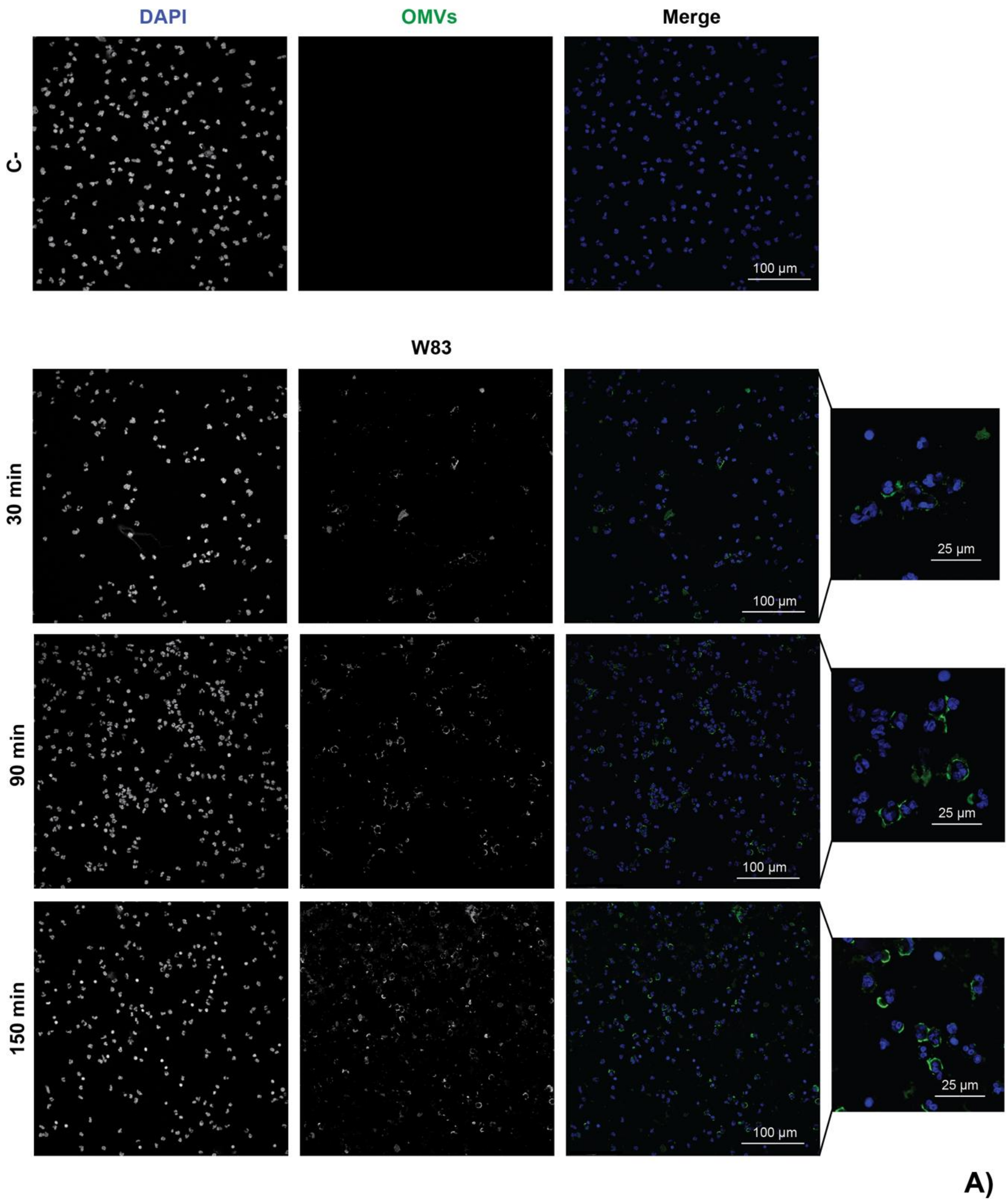

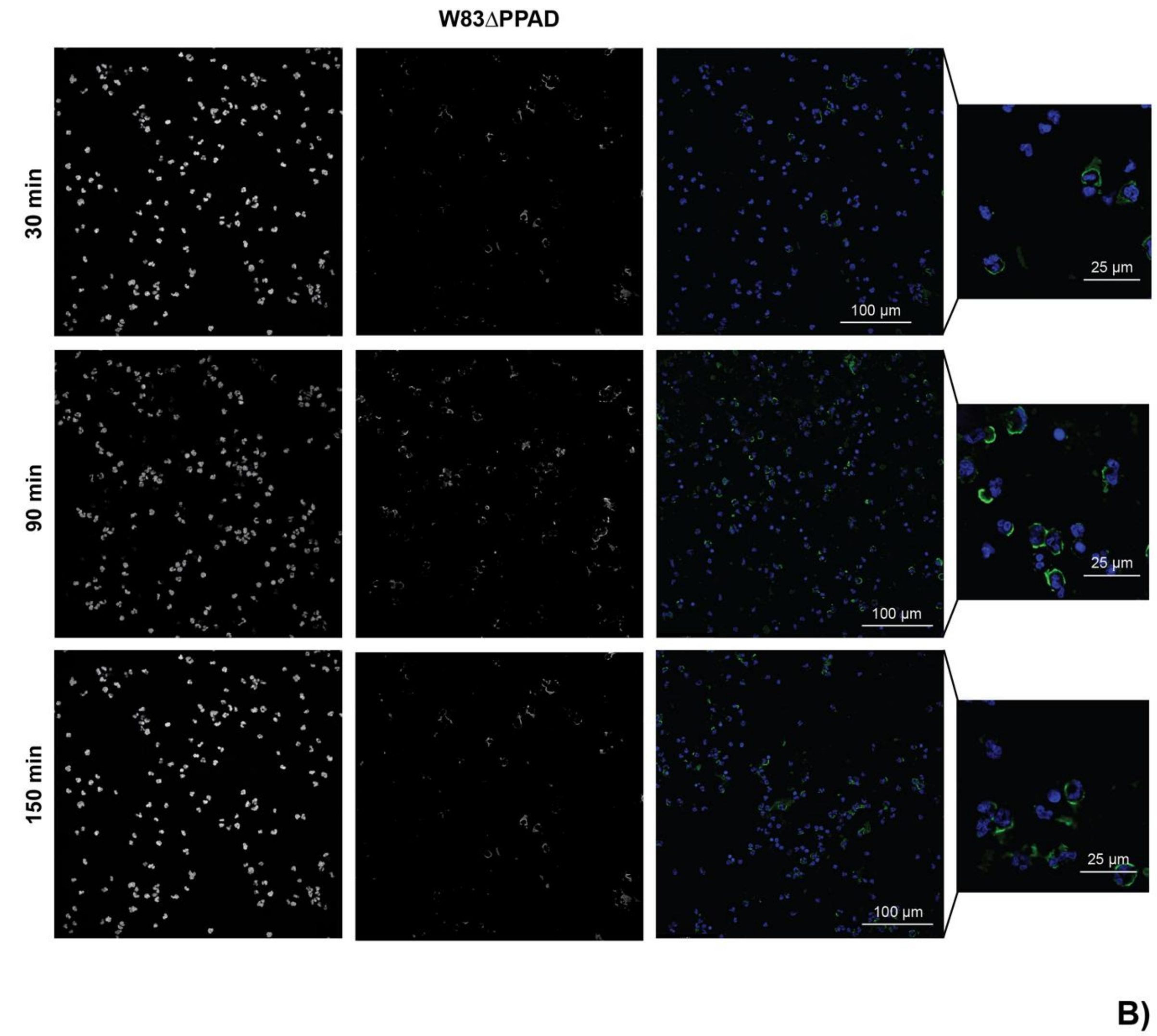

Supplementary Figure S2. Neutrophils do not internalize OMVs of *P. gingivalis* within 150 minutes. (A and B) Confocal fluorescent microscopic images of neutrophils at different time points (30, 90 and 150 min) after addition of 5 µg of W83 OMVs (A) or W83ΔPPAD OMVs (B). DAPI was used to stain the neutrophils' nuclei (blue) and OMVs were labelled using *P. gingivalis*-specific polyclonal rabbit antibodies and secondary goat-anti-rabbit antibodies labelled with AlexaFluor488 (green). Scale bars in the panels with the merged images mark 25 or 100 µm.

Supplementary Figure S3

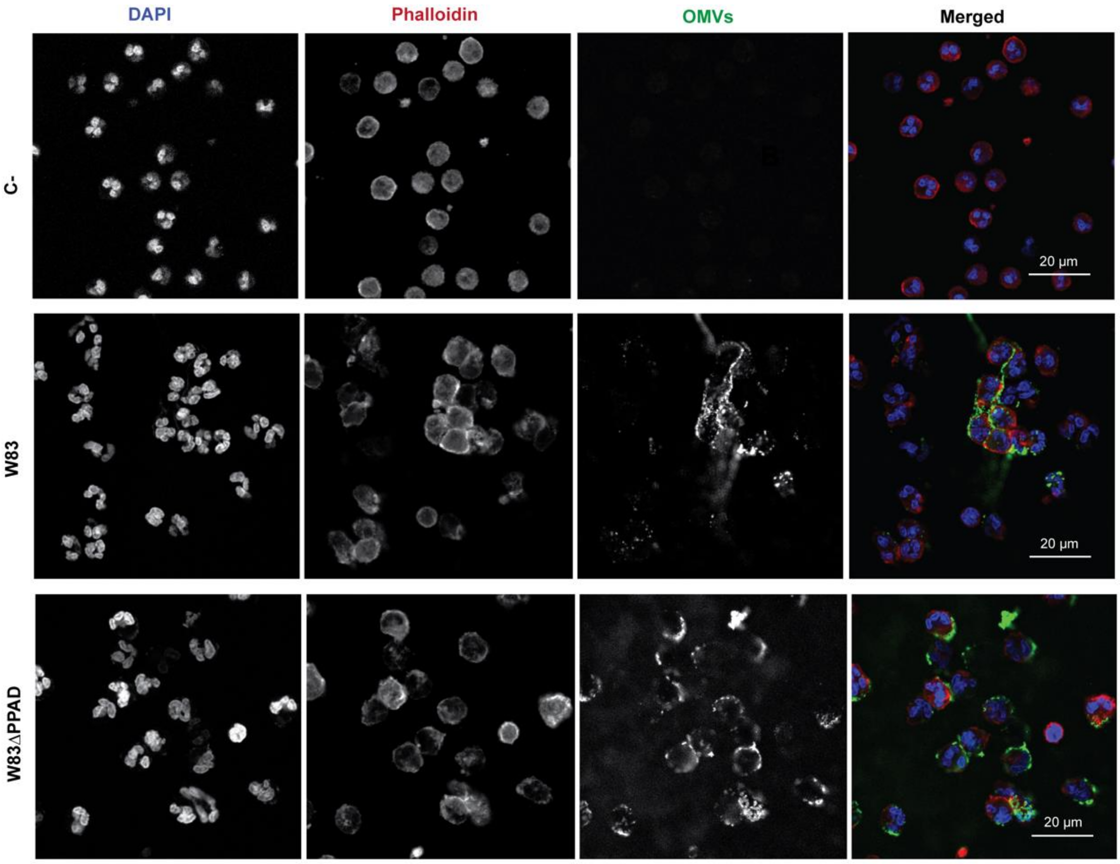

A)

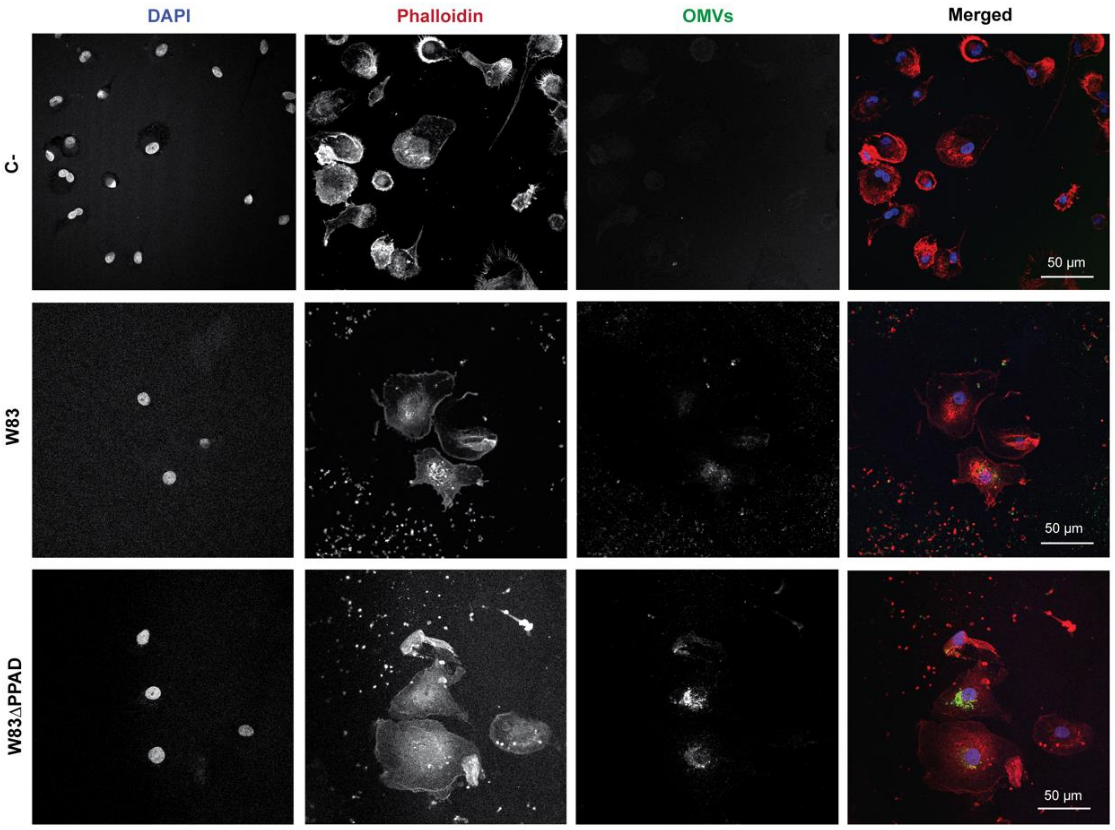

B)

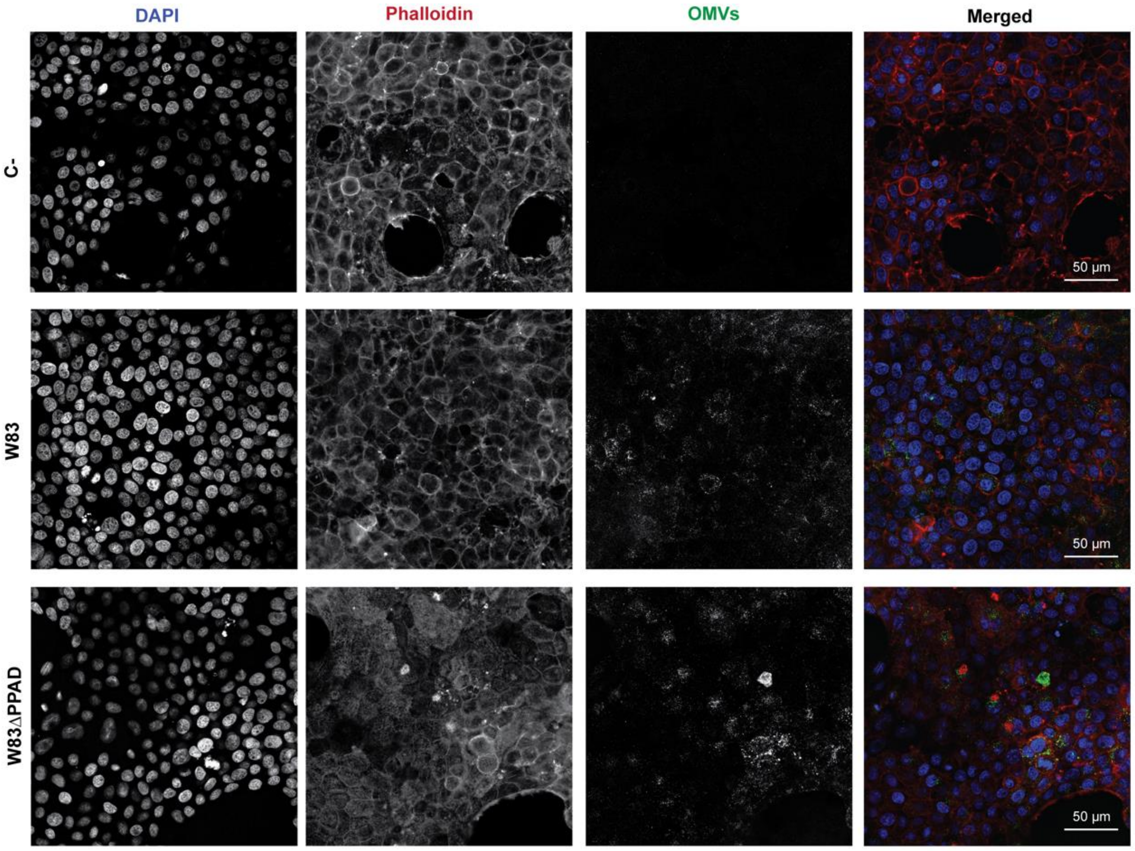

C)

Supplementary Figure S3. Neutrophil, macrophages and A253 cells + OMVs.

(A, B and C) Representative confocal fluorescence microscopy images of neutrophils (A), macrophages (B) and A253 cells (C) challenged with 5 µg of OMVs of *P. gingivalis* W83 or W83ΔPPAD, corresponding to Figure 2 in the main manuscript. DAPI was used to stain the cells' nuclei (blue) and Phalloidin-TRITC (red) was used to stain actin. Additionally, OMVs were labelled with *P. gingivalis*-specific polyclonal rabbit antibodies and secondary goat-anti-rabbit antibodies labelled with AlexaFluor488 (green). Scale bars in the panels with the merged images mark 50 µm.

Supplementary Figure S4

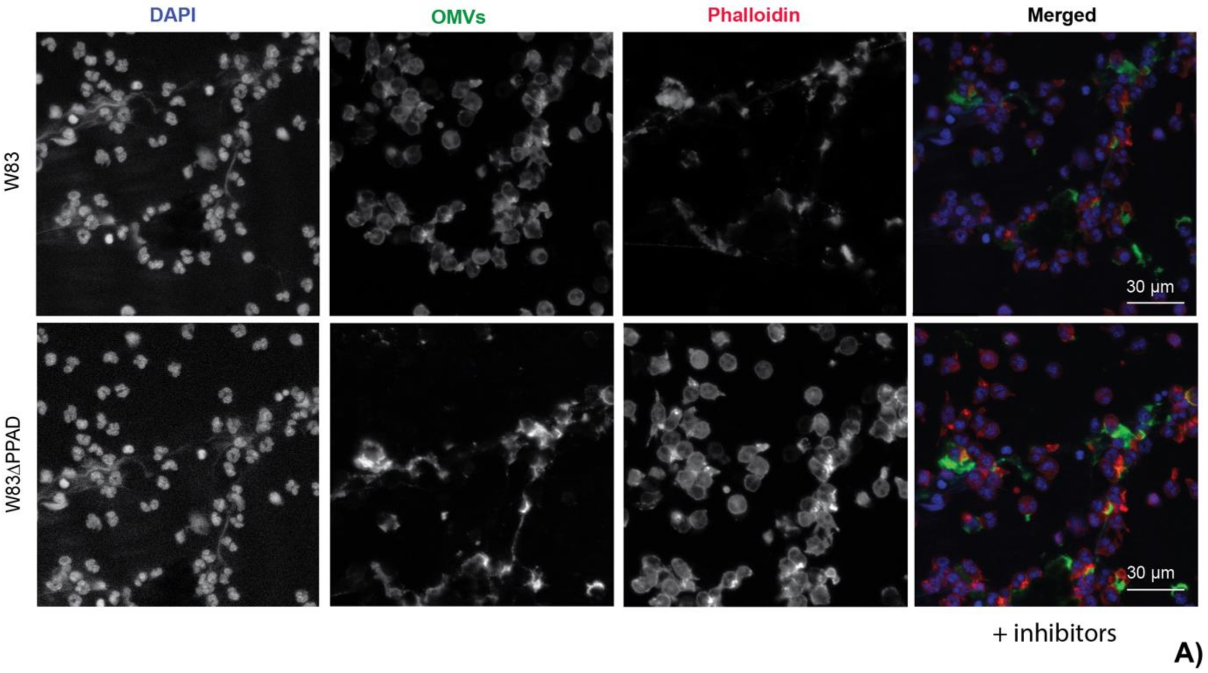

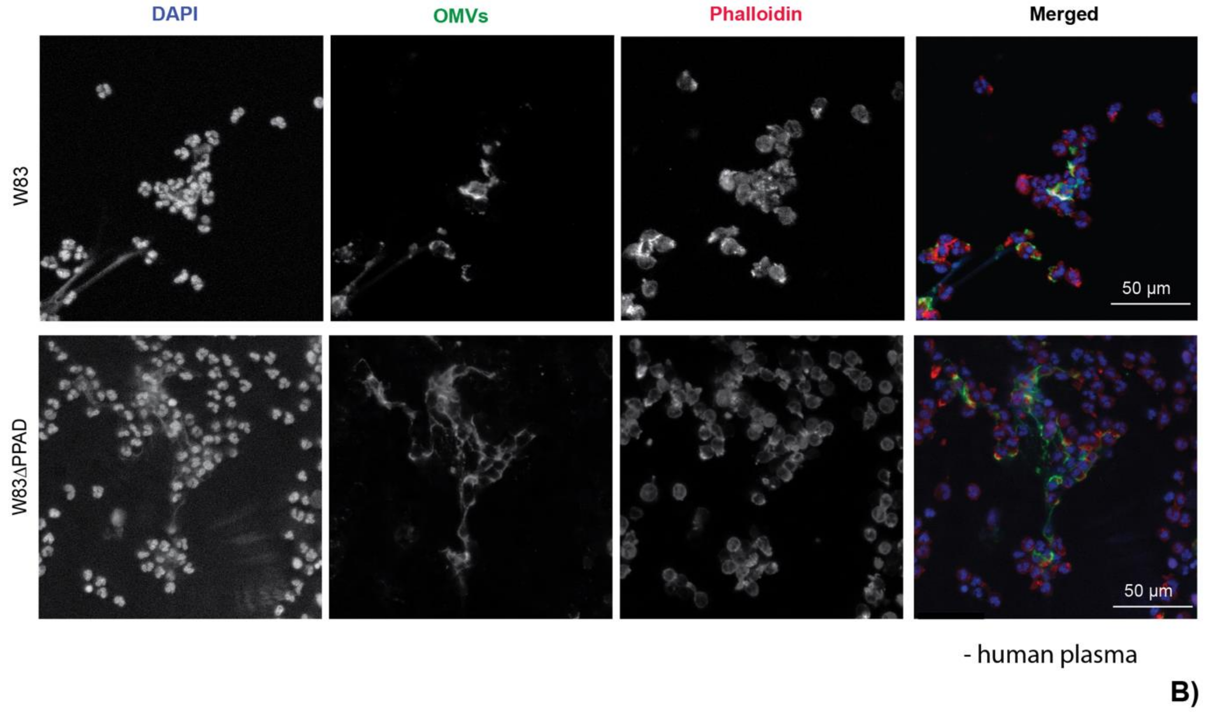

Supplementary Figure S4. OMVs of *P. gingivalis* bind to the neutrophil's surface independently of gingipain activity or the presence of plasma.

(A and B) Confocal fluorescence microscopy images of neutrophils after addition of 5 µg of OMVs isolated from *P. gingivalis* W83 or W83ΔPPAD in the presence of gingipain inhibitors (A) or the absence of human plasma (B). DAPI was used to stain the neutrophils' nuclei (blue) and Phalloidin-TRITC was used to stain actin (red). Additionally, OMVs were labelled with *P. gingivalis*-specific polyclonal rabbit antibodies and secondary goat-anti-rabbit antibodies labelled with AlexaFluor488 (green). Scale bars in the panels with the merged images mark 50 µm.

Supplementary Figure S5

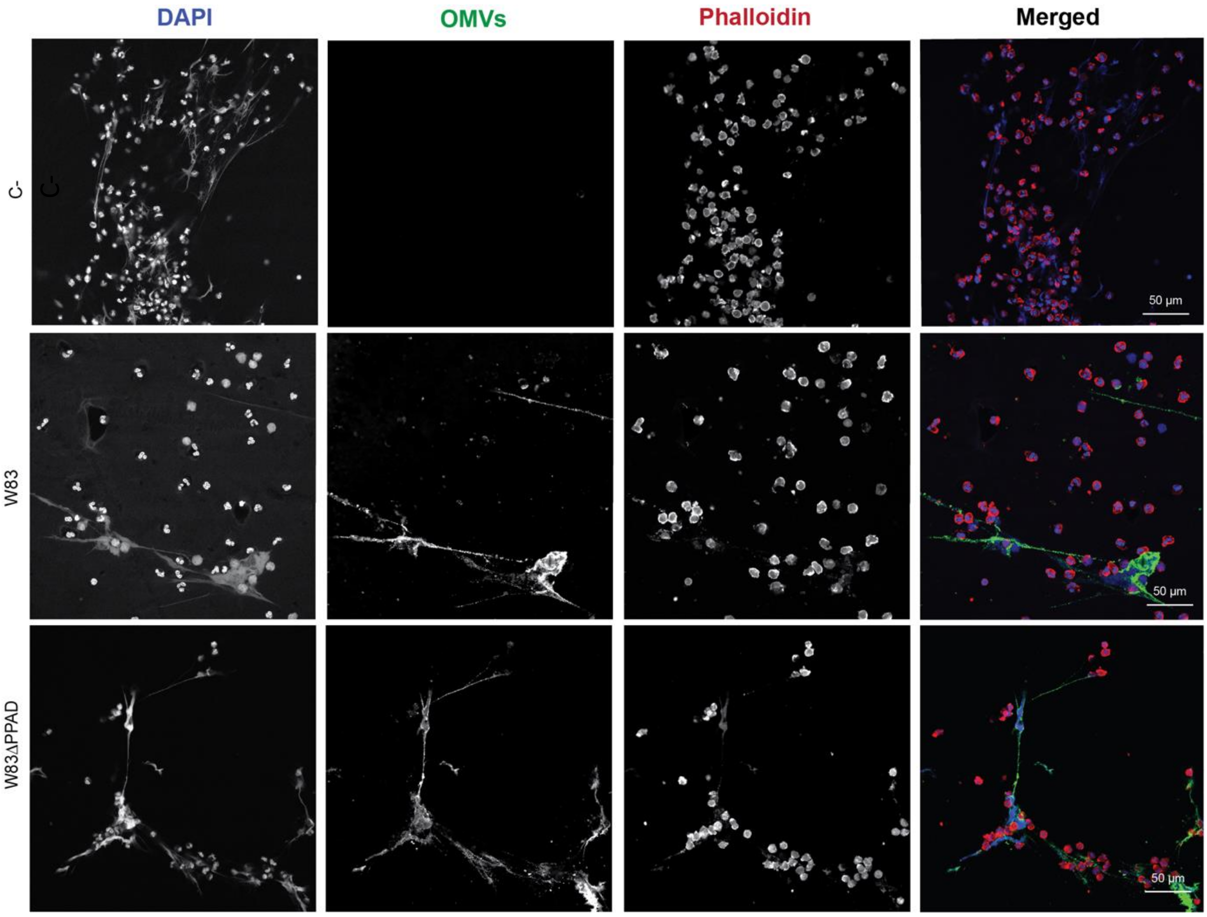

Supplementary Figure S5. OMVs of *P. gingivalis* are trapped in NETs.

(A) Representative confocal microscopy images of 5 μg of OMVs isolated from *P. gingivalis* W83 or W83ΔPPAD trapped in formed NETs. DAPI was used to stain the neutrophils' nuclei and NETs (blue) and Phalloidin-TRITC was used to stain actin (red). Additionally, OMVs were labelled with *P. gingivalis*-specific polyclonal rabbit antibodies and secondary goat-anti-rabbit antibodies labelled with AlexaFluor488 (green). Unchallenged neutrophils were used as a control (C-). Scale bars in the panels with the merged images mark 50 μm. The images in this Figure correspond to Figure 3 in the main manuscript.

Supplementary Figure S6

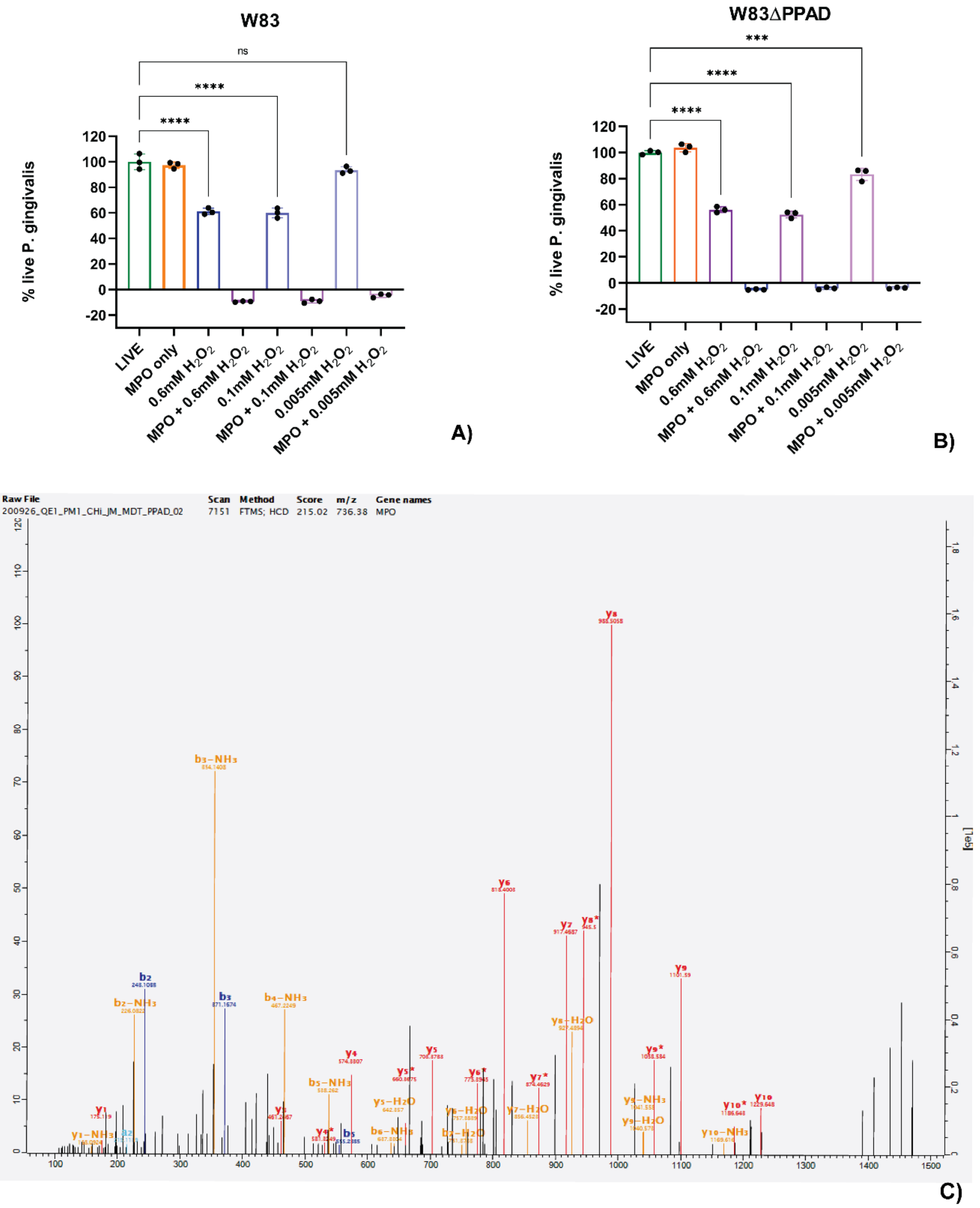

Supplementary Figure S6. OMV-mediated MPO degradation prevents bacterial killing.
(A) MPO-mediated killing of *P. gingivalis* strains W83 or W83ΔPPAD in the presence of different concentrations of H_2O_2, as determined with LIVE/DEAD BacLight Bacterial Viability assay. Note that negative viability values were obtained when the bacteria were incubated with MPO (200 ng) and H_2O_2, which could be attributed to effects of the generated ROS on the dyes applied for the LIVE/DEAD staining. Statistical significance was assessed with an ordinary one-way ANOVA analysis followed by a multiple comparison test to the live bacteria control group: ***, $P \leq 0.001$; ****, $P \leq 0.0001$; ns, not significant. (B) Spectra showing a potential citrullination site on arginine 569 of MPO.

Supplementary Figure S7

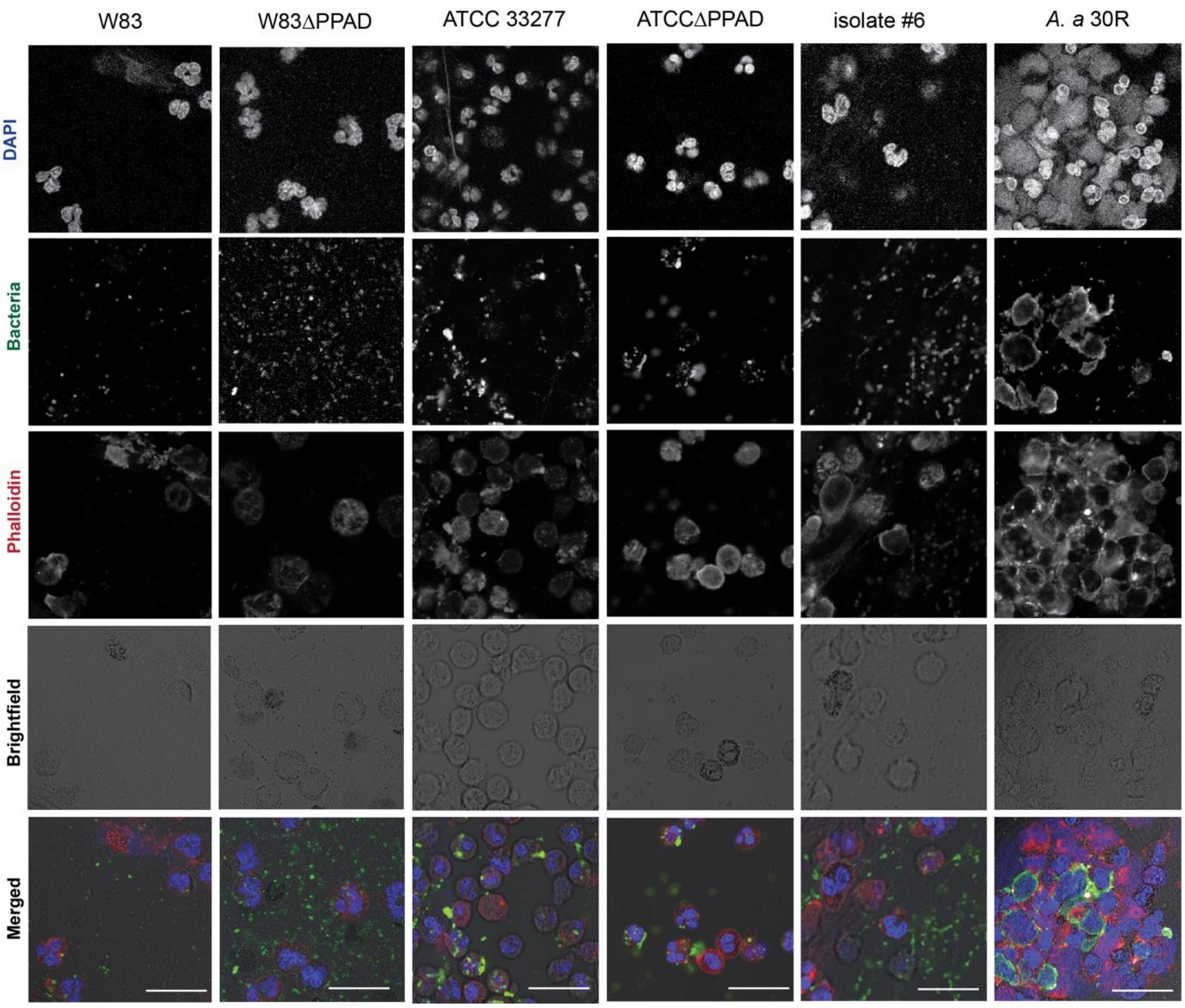

Supplementary Figure S7. Interaction of *P. gingivalis* and *A. actinomycetemcomitans* with human neutrophils.

Representative confocal microscopy images of neutrophils challenged with *P. gingivalis* strains W83, W83ΔPPAD, ATCC 33277 or ATCCΔPPAD, the clinical isolate *P. gingivalis* #6, or *A. actinomycetemcomitans* 30R. DAPI was used to stain the neutrophils' nuclei or extracellular DNA (blue) and Phalloidin-TRITC was used to stain actin (red). Bacterial cells were identified with *P. gingivalis*- or *A. actinomycetemcomitans*-specific polyclonal rabbit antibodies and secondary goat-anti-rabbit antibodies labelled with AlexaFluor488 (green). Scale bars in the panels with the merged images mark 20 µm. The images in this Figure correspond to Figure 7 in the main manuscript.

Supplementary Video S1. Neutrophils challenged with OMVs of *P. gingivalis* W83. Three-dimensional reconstructions from Z-stacks of two-dimensional confocal microscopy images of neutrophils challenged with 5 µg of OMVs of *P. gingivalis* W83. DAPI was used to stain the neutrophils' nuclei (blue). Bacterial OMVs were identified with *P. gingivalis*-specific polyclonal rabbit antibodies and secondary goat-anti-rabbit antibodies labelled with AlexaFluor488 (green).

Supplementary Video S2. Neutrophils challenged with OMVs of *P. gingivalis* W83ΔPPAD. Three-dimensional reconstructions from Z-stacks of two-dimensional confocal microscopy images of neutrophils challenged with 5 µg of OMVs of *P. gingivalis* W83ΔPPAD. DAPI was used to stain the neutrophils' nuclei (blue). Bacterial OMVs were identified with *P. gingivalis*-specific polyclonal rabbit antibodies and secondary goat-anti-rabbit antibodies labelled with AlexaFluor488 (green).

Supplementary Video S3. A253 epithelial cells challenged with OMVs of *P. gingivalis* W83.
Three-dimensional reconstructions from Z-stacks of two-dimensional confocal microscopy images of A253 epithelial cells challenged with OMVs of *P. gingivalis* W83. DAPI was used to stain the epithelial cells' nuclei (blue) and Phalloidin-TRITC was used to stain actin (red). Additionally, OMVs were labelled with *P. gingivalis*-specific polyclonal rabbit antibodies and secondary goat-anti-rabbit antibodies labelled with AlexaFluor488 (green). Note that the Videos 3A and 3B present different angles of the same video.

Supplementary Video S4. A253 epithelial cells challenged with OMVs of *P. gingivalis* W83ΔPPAD.
Three-dimensional reconstructions from Z-stacks of two-dimensional confocal microscopy images of A253 epithelial cells challenged with OMVs *P. gingivalis* W83ΔPPAD. DAPI was used to stain the epithelial cells' nuclei (blue) and Phalloidin-TRITC was used to stain actin (red). Additionally, OMVs were labelled with *P. gingivalis*-specific polyclonal rabbit antibodies and secondary goat-anti-rabbit antibodies labelled with AlexaFluor488 (green). Note that the Videos 4A and 4B present different angles of the same video.

Supplementary Table S1. Mass spectrometry analysis of a granule-derived MPO preparation incubated with or without recombinant PPAD. Granule-derived MPO was incubated overnight at 37 ^{o}C with or without recombinant PPAD. Subsequently, the samples were analyzed by MS. The determined protein LFQ intensities show that MPO was the most abundant protein in the samples and that MPO was identified in all samples. PPAD was only identified with two unique peptides in the 3rd replicate sample. A potential citrullination site was detected on arginine 569 of MPO and manually validated.
